# Prognostic factors of adjuvant chemotherapy discontinuation among stage III colon cancer patients: A survey of medical oncologists and a systematic review and meta‐analysis

**DOI:** 10.1002/cam4.2843

**Published:** 2020-01-21

**Authors:** Devon J. Boyne, Dylan E. O'Sullivan, Emily V. Heer, Robert J. Hilsden, Tolulope T. Sajobi, Winson Y. Cheung, Darren R. Brenner, Christine M. Friedenreich

**Affiliations:** ^1^ Department of Community Health Sciences Cumming School of Medicine University of Calgary Calgary Alberta Canada; ^2^ Department of Cancer Epidemiology and Prevention Research CancerControl Alberta Alberta Health Services Calgary Alberta Canada; ^3^ Department of Public Health Sciences Faculty of Medicine Queen's University Kingston Ontario Canada; ^4^ Department of Medicine Cumming School of Medicine University of Calgary Calgary Alberta Canada; ^5^ Department of Oncology Cumming School of Medicine University of Calgary Calgary Alberta Canada

**Keywords:** adherence, adjuvant chemotherapy, colorectal neoplasms, completion, discontinuation

## Abstract

**Background:**

Factors that are prognostic of early discontinuation of adjuvant chemotherapy among stage III colon cancer patients have yet to be described. To address this gap, a survey of medical oncologists and a systematic review and meta‐analysis were conducted.

**Methods:**

A survey was distributed in March 2019 to medical oncologists who treat colon cancer within Alberta, Canada. Clinicians were asked to rank the prognostic importance of a set of variables using a Likert scale and agreement was quantified using a weighted Cohen's kappa. In addition, we systematically searched four databases up to July 2019. Meta‐analyses were conducted using a random‐effects model.

**Results:**

Of the 25 clinicians who were sent the survey, 14 responded. Overall, there was no agreement regarding which variables were prognostic of early discontinuation (weighted Cohen's kappa = 0.12; 95% CI = 0.05‐0.18). From an initial 3927 articles, 18 investigations were identified for inclusion in our review. Based upon evidence from both the survey and the systematic review, the following four variables were identified as being prognostic of early discontinuation: (a) comorbidity (OR_2+ vs 0_ = 1.53; 95% CI = 1.30‐1.79); (b) performance status (OR_ECOG 2+ vs 0‐1_ = 1.33; 95%CI = 1.07‐1.65); (c) T stage (OR_T4 vs T1‐2_ = 1.57; 95% CI = 0.99‐2.50); and (d) chemotherapy regimen (estimates not pooled due to heterogeneity). In addition to these factors, there was some suggestion that age, marital status/social support, muscle mass, N stage, and tumor grade had prognostic value.

**Conclusions:**

Current evidence is heterogeneous and limited. Additional research is needed to confirm our findings and to explore additional prognostic factors.

## Graphical abstract

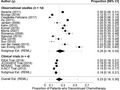

## 1 INTRODUCTION

Among individuals diagnosed with stage III colon cancer, treatment with adjuvant chemotherapy has been shown to increase six‐year survival from approximately 50% to 73%.^1^, ^2^ Despite such benefits, there are side‐effects to adjuvant chemotherapy which have been well‐documented in this patient population. During the course of their treatment, patients often experience a variety of mild to severe toxicities which include neuropathy, diarrhea, nausea, vomiting, fatigue, and hand‐foot syndrome.^3^, ^4^, ^5^ In the recent IDEA Trial (2018), for example, which assessed the effect of a shortened duration of adjuvant chemotherapy on disease‐free survival among individuals with stage III colon cancer, one in two patients in the 6‐month duration arm experienced at least one grade 3 or 4 adverse event.^4^ As a result of such toxicities, some patients willingly or are mandated by their oncologist to discontinue their prescribed chemotherapy regimen prematurely which may compromise the efficacy of adjuvant treatment. In addition to treatment side‐effects, non‐medical factors may contribute an individual's willingness to complete chemotherapy such as barriers related to treatment costs and ease of access. Other less common medical reasons for discontinuation of adjuvant chemotherapy include disease progression and death.

Variables that are prognostic of early discontinuation of adjuvant chemotherapy among stage III colon cancer patients have not been systematically described and quantified. Previous reviews have focused on qualitatively synthesizing results from investigations targeting breast cancer patients,^6^, ^7^ elderly cancer patients,^8^, ^9^ and patients prescribed oral antineoplastic medications.^6^, ^10^ While these studies have reported high percentages of patients discontinuing chemotherapy and have identified potential prognostic factors of early discontinuation,^6^, ^7^, ^8^, ^9^, ^10^ such findings may not be generalizable to patients with stage III colon cancer. The identification of prognostic factors for early discontinuation among stage III colon cancer patients may help to improve clinical practice and research. Such an understanding may, for example, help to inform the appropriateness of treatment and the intensity of follow‐up care. The identification of prognostic factors could also help in the development of clinical prediction models by narrowing the list of candidate variables. In addition, this knowledge may help to identify potential confounders which would assist with the adjustment for confounding in etiologic studies examining the association between chemotherapy duration and patient outcomes. In a previous review of the association between chemotherapy duration and survival, we recently identified the need for such etiologic research in order to validate subgroup findings from the IDEA Trial (2018) and highlighted the lack of adjustment for potential confounders as a limitation of many of the observational studies conducted to date.^11^

To address this gap, we surveyed the opinions of medical oncologists and conducted a systematic review and meta‐analysis in order to identify variables that were prognostic of adjuvant chemotherapy discontinuation. The purpose of this study was to provide evidence that would support: (a) the development of clinical tools used to predict the risk of discontinuation at the patient‐level by helping to narrow a list of candidate variables and; (b) the estimation of the effect of chemotherapy duration on patient outcomes within observational settings by helping to identify a list of potential baseline confounders that should be considered in the analyses. By examining both sources of evidence, we hoped to increase our certainty regarding variable importance and better identify areas for future research. The specific objectives of this investigation were to: (a) survey a group of medical oncologists and describe their perceptions regarding the prognostic importance of variables with respect to chemotherapy discontinuation; (b) determine if there was any agreement between clinicians regarding objective one; (c) systematically review the evidence to date and quantify the association between various patient, tumor, treatment, and provider characteristics and chemotherapy discontinuation; and (d) estimate the proportion of patients with stage III colon cancer who discontinue adjuvant chemotherapy.

## 2 METHODS

### 2.1 Survey of medical oncologists in Alberta, Canada

A cross‐sectional survey was conducted to assess the opinions of practicing medical oncologists within Alberta, Canada regarding the prognostic importance of variables of early chemotherapy discontinuation. The survey was distributed anonymously online to medical oncologists who treat colon cancer at the Tom Baker Cancer Centre in Calgary, Alberta (n = 9), the Cross Cancer Centre in Edmonton, Alberta (n = 9), and at tertiary centers within communities across Alberta (n = 7). The survey was first distributed in March 2019 and a follow‐up survey was sent in May 2019. In the survey, clinicians were presented with a list of variables and were asked to state if the variable was “not”, “somewhat”, “very”, or an “extremely” important prognostic factors for chemotherapy discontinuation among stage II or III colon cancer patients or if they were “unsure”. The variables that were included in the survey were: age, biological gender, body mass index, education, household income, rural residence, previous diagnosis of cancer, cardiovascular disease, diabetes, or chronic pulmonary disease, T stage, N stage, tumor grade, right‐sided tumor, baseline laboratory results, time from surgery to chemotherapy initiation, and the number of lymph nodes examined. Clinicians were also asked to list variables that were important prognostic factors and were not listed in the survey.

This survey was originally intended to guide the selection of variables for a separate study in which we developed a clinical tool for predicting chemotherapy discontinuation. The list of variables included in the survey was limited to variables that were routinely and reliably captured within the administrative datasets that would support this project. For this reason, we did not include variables such as performance status within our survey. In addition, we did not include type of chemotherapy within our survey because we had decided to include this variable within the clinical prediction model a priori.

### 2.2 Statistical analysis of survey responses

Overall agreement between clinicians was assessed using the model‐based method of estimating the weighted Cohen's kappa developed by Nelson and Edwards (2015).^12^ This method can be used to assess the degree of agreement between three or more raters when the outcome is ordinal. The variables were also examined using two summary scores: (a) the percentage of clinicians who classified the variable as being at least “Somewhat Important”; and (b) the median response. In all analyses, responses coded as “Unsure” were treated as missing values.

### 2.3 Systematic search and study eligibility

The systematic review and meta‐analysis were conducted following the guidance of the Cochrane Handbook^13^ and Riley et al (2019)^14^ and reported in accordance with the PRISMA statement.^15^ The protocol used for this study was adopted from an existing protocol used for a separate investigation which examined the association between a shortened duration of adjuvant chemotherapy and survival among individuals with stage II or III colon cancer (PROSPERO identifier of original protocol: CRD42018108711).^11^ The search strategy used for the current review was originally created for and is available in the supplemental file of the systematic review on chemotherapy duration and survival that we previously published.^11^ The original search was conducted on August 10th, 2018, and covered English abstracts in the MEDLINE, EMBASE, CENTRAL, and CINAHL databases. The database search was re‐run on July 8th, 2019 to identify studies published since the date of the original search.

Given our focus on prognostic factors, we adopted the PICOTS modification of the PICO mnemonic when developing our research question and eligibility criteria.^14^ The Population of interest was individuals with stage III colon cancer who underwent surgical resection of their tumor and were prescribed adjuvant chemotherapy in the form of 5‐flourouracil or capecitabine alone or in combination with oxaliplatin. We placed no restriction on the Index prognostic factors or the Comparator prognostic factors (ie other prognostic factors included in a multivariable model) which could consist of any patient, clinician, tumor, treatment, or health‐system variables. The Outcome of interest was the risk or odds of discontinuation of adjuvant chemotherapy defined in part or entirely by the number of cycles or months of treatment completed by the patient. With respect to Timing our interest was in variables measured prior to the initiation of adjuvant chemotherapy that could be used to predict discontinuation during the course of their prescribed treatment regimen. We made no restrictions on the clinical Setting in which individuals were administered adjuvant chemotherapy (eg tertiary, community, academic, private, etc).

All articles were reviewed by DJB and EH for eligibility. The title and abstract of each study were first assessed for relevance and then the full‐texts of the remaining studies were examined. Any disagreements were resolved through discussion. We excluded articles if they: (a) were review articles, case studies, or case series; (b) were not conducted on human participants; (c) did not assess the association between any patient, tumor, provider, or contextual variables and the odds or risk of adjuvant chemotherapy discontinuation; (d) did not include patients with stage III colon cancer; (e) did not explore treatment discontinuation defined according to the duration of 5‐flourouracil or capecitabine alone or in combination with oxaliplatin; and (f) had a sample size that was less than 200 individuals. A minimum sample size of 200 was chosen because approximately 200 individuals are needed to estimate the proportion of patients who discontinue chemotherapy within two exposure categories with 95% confidence limits that are within ± 10 percentage points of the point estimate which we felt was the minimal degree of precision needed for an estimate to be clinically informative.^16^ We chose to examine the complete cessation of adjuvant chemotherapy as an outcome rather than periodic dose‐reductions or dose‐omissions in order to focus on the form of non‐adherence with the greatest potential to impact treatment efficacy.

### 2.4 Meta‐analysis

All meta‐analyses were conducted using a random‐effects model estimated using restricted maximum likelihood (REML). For objective three, the log odds ratio was estimated for each study and the natural exponential function was used to convert the pooled results back to the original scale. When a study reported stratified results, the estimates were pooled such that each study contributed a single estimate in the meta‐analysis. When multiple measures of effect were reported, we used the estimate adjusted for the greatest number of variables. When synthesizing the association between age and the odds of discontinuation, a log‐linear association was first estimated for each study using the Greenland‐Longnecker method prior to the quantitative synthesis in a “two‐stage” approach.^17^ If it was not reported, the median value of the different age categories was taken as the midpoint (eg midpoint of 65‐75 year category = 70 years). If the maximum and minimum values of an age category were not reported, we the distance between intermediary categories to estimate the median value of the upper or lower‐most categories (eg 65‐70, 70‐75, 75‐80, 80 + year categories; we would estimate a median of 82.5 years for the 80 + year category). In cases where a meta‐analysis was not possible (ie there was only one estimate) or inappropriate because of heterogeneity, then results were qualitatively described. For objective four, the log odds of discontinuation was first estimated within each study and the inverse logit transformation was used to return the pooled estimate as a proportion. For all meta‐analyses, heterogeneity was assessed using the tau‐squared, I‐squared, and 95% prediction intervals.^18^, ^19^ Publication bias was examined using a funnel plot. In accordance with recommendations in the Cochrane Handbook, we did not examine publication bias within meta‐analyses involving less than 10 studies.^13^

### 2.5 Risk of BIAS

Risk of bias was assessed independently and in duplicate by DJB and DEO using the Quality in Prognostic Factor Studies (QUIPS) tool as per the recommendations of Riley et al (2019).^14^, ^20^ Discrepancies were resolved through discussion. With respect to the “prognostic factor measurement” domain, we assigned a “moderate” risk of bias to studies that dichotomized one or more continuous exposure variables because this practice is known to be statistically problematic.^21^ Regarding the “adjustment for other prognostic factors” domain, we assigned a “high” risk of bias to studies presenting crude estimates, a “moderate” risk of bias to studies in which there were four or fewer variables in total within the multivariable model, and a “low” risk of bias to studies that included five or more factors within a multivariable model or to studies where participants were randomly assigned to different exposure categories. When assessing the risk of bias within this domain, we did not identify a minimal adjustment set of covariates because no factors to date have been established as being prognostic of chemotherapy discontinuation within this patient population. Specific to the “statistical analysis and reporting” domain, we assigned a “moderate” risk of bias to studies in which there was evidence that the reporting of results was related to the precision or the statistical significance of the estimates. No additional criteria beyond those described in the QUIPS tool were used to assess the risk of bias within the remaining domains.

### 2.6 Software

Statistical analyses were conducted using R Studio version 1.1.463.^22^ The R code used to carry out the estimation of the weighted kappa statistic was taken from the supplemental file of Mitani et al (2017).^23^ Meta‐analyses were conducted using the *metafor* package^24^ and the Greenland‐Longnecker method was implemented using the *dosresmeta* package in R Studio.^25^

## 3 RESULTS

### 3.1 Survey of medical oncologists

Of the 25 medical oncologists contacted, 14 (56%) responded to our survey. Overall, there was no agreement between clinicians beyond what would be expected due to chance alone (weighted Cohen's kappa = 0.12; 95% CI: 0.05‐0.18). Age was the only variable where 100% of clinicians thought that it was at least “somewhat important” and where the median response was “very important” (Table 1). Besides age, 70% or more of the clinicians thought that the following variables were at least “somewhat important” prognostic factors for chemotherapy discontinuation: (a) a history of cardiovascular disease, cancer, or diabetes; (b) time from surgery to chemotherapy initiation; (c) baseline laboratory values; and (d) T and N stage. The majority of clinicians were of the opinion that the following variables were “not important” prognostic factors of chemotherapy discontinuation: (a) body mass index; (b) gender; and (c) tumor side. The following is a list of additional variables not included in our survey that were considered by one or more clinician to be an important prognostic factors: (a) use of or interest in alternative medicines; (b) social support; (c) health literacy; (d) performance status; (e) the type of chemotherapy regimen; (f) body composition and muscle mass; (g) distance from home to treatment facility; (h) mental health; (i) post‐operative complications; and (j) religious or spiritual beliefs.

**Table 1:** Results from survey of medical oncologists (n = 14) in Alberta, Canada

Variable	At Least “Somewhat Important” (%)	Variable importance (No. of Responses)					
		Median Response	Not	Somewhat	Very	Extremely	Unsure
Age	100.0	Very	0	6	5	3	0
History of CVD	85.7	Somewhat	2	9	2	1	0
Time from surgery to chemotherapy initiation	85.7	Somewhat	2	7	2	3	0
History of cancer	78.6	Somewhat	3	10	1	0	0
History of diabetes	78.6	Somewhat	3	10	1	0	0
Laboratory values	71.4	Somewhat	4	6	4	0	0
N Stage	71.4	Somewhat	4	4	4	2	0
T Stage	71.4	Somewhat	4	6	2	2	0
Urban/Rural Residence	61.5	Somewhat	5	6	2	0	1
History of COPD	57.1	Somewhat	6	7	1	0	0
Number of lymph nodes examined	57.1	Somewhat	6	4	2	2	0
Education	53.8	Somewhat	6	5	1	1	1
Tumor Grade	53.8	Somewhat	6	5	1	1	1
Income	53.8	Somewhat	6	6	1	0	1
Body Mass Index	35.7	Not	9	5	0	0	0
Gender	28.6	Not	10	4	0	0	0
Tumor Side	21.4	Not	11	1	1	1	0

### 3.2 Systematic review

**Figure 1:**
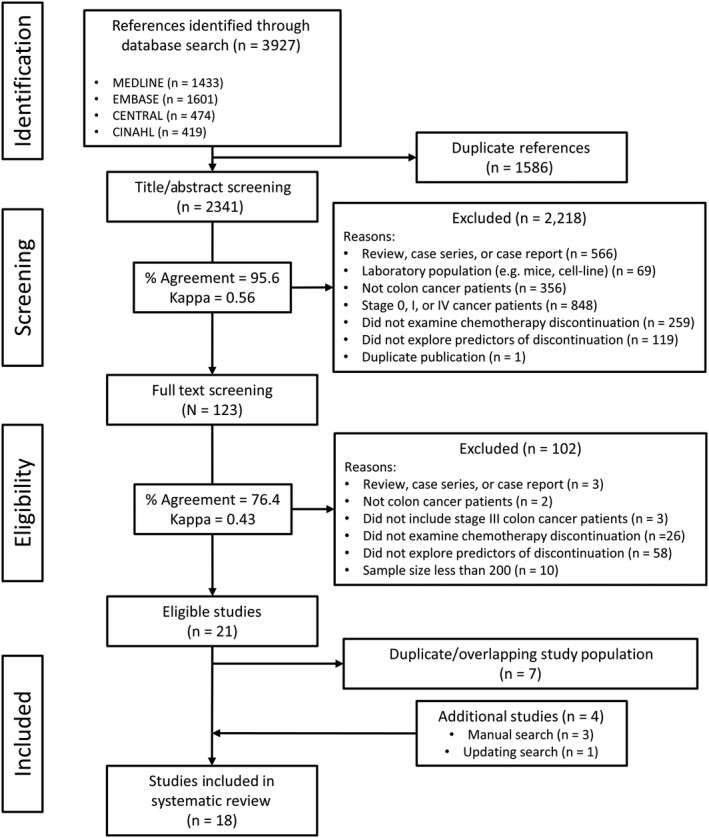
PRISMA flow diagram. This figure describes the inclusion and exclusion of studies in our systematic review

**Figure 2:**
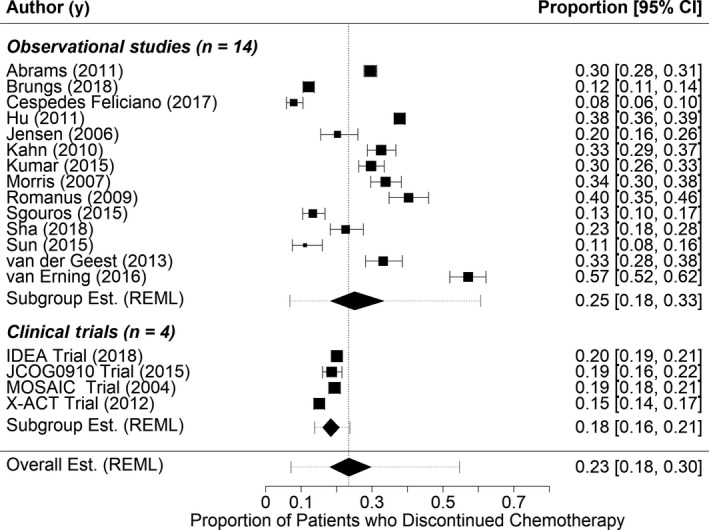
The proportion of stage III colon cancer patients who discontinued chemotherapy among studies included in systematic review (n = 18). This figure presents results from a meta‐analysis of the proportion of patients who discontinued chemotherapy stratified by study design

From an initial 3927 articles, 18 investigations were identified for inclusion in our review (14 observational studies and four randomized trials) (Figure 1 and Table 2).^3^, ^4^, ^5^, ^26^, ^27^, ^28^, ^29^, ^30^, ^31^, ^32^, ^33^, ^34^, ^35^, ^36^, ^37^, ^38^, ^39^, ^40^ Among the observational studies, the pooled estimate of the proportion of patients who discontinued chemotherapy was 25.1% (95% CI: 18.3%‐33.3%) (Figure 2). There was substantial heterogeneity in these estimates, likely arising from the disparity in the definitions of chemotherapy discontinuation (*I* ^2^: 98.8%; tau^2^: 0.567; 95% prediction interval: 6.8%‐60.7%). Among the four randomized trials included in this review, the pooled proportion of patients who discontinued was 18.3% (95% CI: 16.0%‐20.7%; *I* ^2^: 89.6%; tau^2^: 0.148; 95% prediction interval: 13.8%‐23.7%) (Figure 2).

**Table 2:** Overview of studies included in systematic review of determinants of chemotherapy discontinuation among stage III colon cancer patients (n = 18)

Study	Country	Study population	Period of diagnosis	No. patients	No. events (%)	Regimen(s)	Objective to identify predictors of discontinuation^a^	Definition of chemotherapy discontinuation	Prognostic factors examined
Cohort studies									
Abrams (2011)^26^	United States	Stage II/III Colon Cancer	2004 to 2010	2501	738 (29.5)	5FU, CapMono, FOLFOX, CAPOX	Yes	Less than 3 mo	Age, ECOG, oncologist case volume, gender, treatment facility, tumor stage, and type of chemotherapy
Brungs (2018)^27^	Australia	Stage III Colon Cancer	2006 to 2013	1626	196 (12.1)	5FU, CapMono, FOLFOX, CAPOX	No	Less than 3 mo	Age
Cespedes Feliciano (2017)^28^	United States	Stage II/III Colon Cancer	2006 to 2011	533	42 (7.9)	FOLFOX	No	Less than 6 cycles	Muscle mass
Hu (2011)^29^	United States	Stage III Colon Cancer Aged ≥ 65 y	1991 to 2005	4660	1761 (37.8)	5FU, CapMono, FOLFOX, CAPOX	Yes	Less than 5 mo in the 1996‐2005 cohort; Less than 8 mo in the 1991‐1995 cohort	Age, comorbidity, marital status, N stage, race, gender, SES, tumor grade, urban/rural
Jensen (2006)^30^	Denmark	Stage III Colon Cancer	1996 to 2003	227	46 (20.3)	5FU	No	Number of cycles (count: 1 to 6); Operationalized as < 6 cycles	Age
Kahn (2010)^31^	United States	Stage III Colon Cancer	2003 to 2005	513	167 (32.6)	5FU, CapMono, FOLFOX, CAPOX	No	Months of chemotherapy (count: 1 to 5); Operationalized as < 5 mo	Age
Kumar (2015)^32^	Canada	Stage III Colon Cancer	2006 to 2010	616	183 (29.7)	FOLFOX	Yes	Less than 10 cycles	Age, comorbidity, ECOG, lymphovascular invasion, N stage, nodes removed, obstruction/perforation, perineural invasion, postoperative stay, T stage, tumor grade, tumor side, time to chemotherapy, gender
Morris (2007)^33^	Australia	Stage III Colon Cancer	1994 to 2001	461	156 (33.8)	5FU	Yes	Less than 4 cycles	Age, lymphocytic response, lymphovascular invasion, perineural invasion,, mucinous, N stage, perforation, preoperative colonoscopy or sigmoidoscopy, SES, gender, surgical case volume, T stage, treatment facility, tumor grade, tumor side,
Romanus (2009)^34^	United States	Stage II/III Colon Cancer	2005 to 2007	293	118 (40.3)	FOLFOX	Yes	Less than 12 cycles	Age, history of diabetes, tumor stage
Sgouros (2015)^35^	Greece	Stage II/III Colorectal Cancer	1995 to 2011	451	60 (13.3)	5FU, FOLFOX, FOLFIRI, Other	No	Failure to complete all cycles	Tumor stage
Sha (2018)^36^	Canada	Stage II/III Colon Cancer	2011 to 2014	306	69 (22.5)	FOLFOX, CAPOX	No	Failure to complete all cycles	Type of chemotherapy
Sun (2015)^37^	Canada	Stage I‐III Colorectal Cancer	2008 to 2012	217	24 (11.1)	CapMono	No	Less than 8 cycles	Age^b^, ECOG, gender, tumor stage
van der Geest (2013)^38^	Netherlands	Stage III Colon Cancer	2006 to 2008	317	105 (33.1)	FOLFOX, CAPOX, Tegafur, CapMono	Yes	Less than 24 wks or 21 wks from first to last cycle and less than 12 cycles of FOLFOX or 8 cycles of CapMono/CAPOX	Age, comorbidity, prolonged hospital stay, reoperation, SES, gender, surgical procedure, tumor grade, tumor side, tumor stage, urgency of surgery
van Erning (2016)^39^	Netherlands	Stage III Colon Cancer	2005 to 2012	357	204 (57.1)	CAPOX, CapMono	Yes	Less than 8 cycles	Age, ASA score, comorbidity, N stage, gender, T stage, tumor grade, tumor side, type of chemotherapy
Randomized clinical trials									
IDEA Trial (2018)^4^	Multiple	Stage III Colon Cancer	2007 to 2015	12 834	2575 (20.1)	CAPOX, FOLFOX	No	Failure to complete all cycles	Type of chemotherapy (not randomized)
JCOG0910 Trial (2015)^40^	Japan	Stage III Colorectal Cancer	2010 to 2013	774	144 (18.6)	CapMono	Yes	Failure to complete all cycles	Age, surgical procedure
MOSAIC Trial (2004)^3^	Multiple	Stage II/III Colon Cancer	1998 to 2001	2247	436 (19.4)	5FU, FOLFOX	No	Less than 12 cycles	Type of chemotherapy
X‐ACT Trial (2012)^5^	Switzerland	Stage III Colon Cancer	1998 to 2001	1967	296 (15.0)	CapMono, 5FU	No	Failure to complete all cycles	Type of chemotherapy

Abbreviations: ASA, American Society of Anesthesiologists; ECOG, Eastern Cooperative Oncology Group.

a Indicator for whether or not one of the study objectives was to examine the association between multiple potential prognostic variables and chemotherapy discontinuation

b The age variable was not included in the synthesis as we were unable to extract an estimate suitable for meta‐analysis

Of the 18 studies included, eight had a specific objective to examine the association between multiple potential prognostic factors and chemotherapy discontinuation (Table 2). In general, there were little to no missing covariate data within the studies included in this review (Table S1). Nine of the 18 studies analyzed the data using multivariable logistic regression whereas crude, strata‐specific proportions were reported for the other nine studies (Table S1). With respect to multivariable adjustment, only one study used backwards elimination with a liberal p‐value to select which subset of covariates to include in the multivariable model (Table S1). The other investigations that reported a multivariable‐adjusted analysis included all covariates that were examined with the exception of Cespedes Feliciano et al (2017) which adjusted for three variables based upon their expert opinion (Table S1).^28^ All of the observational cohort studies collected data retrospectively using medical records and/or routinely collected administrative data (eg hospital or pharmacy dispensary records). The exception to this rule was the study by Cespedes Feliciano (2017) which also used prospectively collected muscle mass data assessed via a computed tomography scan.^28^

The following sections detail results from the meta‐analyses (Table 3). A forest plot for each individual meta‐analysis is available in the supplemental (Figures S1‐S17). Following this section is a narrative summary of results for variables not included in the quantitative synthesis.

**Table 3:** Results from meta‐analysis of individual variables

Variable	No. of Studies	No. of Patients	I^2^	Tau^2^	OR (95% CI)	95% Prediction Interval
Patient variables						
Age (per 10 year increase)	11	12 345	86.86%	0.0599	1.35 (1.14‐1.59)	0.81‐2.34
Gender (female vs male)	7	9129	79.68%	0.0861	1.23 (0.94‐1.61)	0.65‐2.32
Socioeconomic status (lowest vs highest)	3	5438	67.61%	0.3498	1.22 (0.75‐1.98)	0.53‐2.82
Comorbidity		5945				
1 vs 0 comorbidities	3		74.64%	0.1451	0.97 (0.59‐1.61)	0.39‐2.39
2 + vs 0 comorbidities	4		0.00%	0.0000	1.53 (1.30‐1.79)	1.30‐1.79
ECOG score (2 + vs 0‐1)	3	2784	0.00%	0.0000	1.33 (1.07‐1.65)	1.07‐1.65
Tumor characteristics						
Stage (III vs II)	4	3462	0.00%	0.0000	0.96 (0.80‐1.16)	0.80‐1.16
T stage		1434				
T3 vs T1‐2	3		0.00%	0.0000	1.32 (0.90‐1.94)	0.90‐1.94
T4 vs T1‐2	3		0.00%	0.0000	1.57 (0.99‐2.50)	0.99‐2.50
N Stage (N2 vs N1)	4	6094	63.03%	0.0455	1.21 (0.92‐1.59)	0.74‐1.99
Tumor side (right vs left)	4	1751	43.60%	0.0498	1.11 (0.80‐1.55)	0.64‐1.92
Tumor grade (high vs low)	4	5871	0.00%	0.0000	1.29 (1.14‐1.47)	1.14‐1.47
Lymphovascular invasion (yes vs no)	2	1077	0.00%	0.0000	0.97 (0.73‐1.30)	0.73‐1.30
Perineural invasion (yes vs no)	2	1077	0.00%	0.0000	1.11 (0.75‐1.64)	0.75‐1.64
Treatment factors						
Treatment facility		2962				
Community vs academic	2		58.66%	0.1114	1.70 (0.95‐3.08)	0.71‐4.12
Private vs academic	2		0.00%	0.0000	1.21 (0.90‐1.61)	0.90‐1.61
Prolonged post‐operative hospital stay (yes vs no)	2	933	73.47%	0.2985	1.70 (0.72‐4.04)	0.43‐6.66

### 3.3 Meta‐analysis

#### 3.3.1 Patient characteristics

Results from the meta‐analysis suggested that the odds of discontinuation was higher among patients who had two or more comorbidities vs none (OR: 1.53; 95% CI: 1.30‐1.79) and among patients who had an ECOG score of two or greater vs zero or one (OR: 1.33; 95% CI: 1.07‐1.65) (Table 3). Although the meta‐analysis suggested that being older and being female were associated with an increased odds of discontinuation, the evidence base was heterogeneous and the 95% prediction intervals included values below the null. There was no evidence of an association between socioeconomic status and chemotherapy discontinuation.

#### 3.3.2 Tumor variables

There was evidence that the odds of discontinuation was higher among patients with a high vs low grade tumor (OR: 1.29; 95% CI: 1.14‐1.47) and among patients with a T4 vs T1‐2 stage tumor (OR: 1.57; 95% CI: 0.99‐2.50). There was no evidence that discontinuation was associated with tumor stage III vs II or N2 vs N1 or the presence of lymphovascular or perineural invasion (Table 3).

#### 3.3.3 Provider‐related factors

There was some suggestion in the meta‐analysis that patients who received treatment in a community centre instead of an academic centre and that patients who had a prolonged post‐operative hospital stay had an increased odds of discontinuation, however, there was a great deal of imprecision in these estimates and the 95% prediction intervals included values below 1.00 (Table 3).

### 3.4 Narrative summary

#### 3.4.1 Patient characteristics

There were reports from individual studies of a higher odds of discontinuation among individuals who were white vs black (OR: 1.48; 95% CI: 1.12‐1.95),^29^ unmarried vs married (OR: 1.55; 95% CI: 1.21‐1.97),^29^ and in lower vs upper tertiles of muscle mass (OR: 2.34, 95% CI: 1.04‐5.24).^28^ Hu et al (2011) found that living in a rural location was not associated with chemotherapy discontinuation (OR_rural vs big metro_: 1.25; 95% CI: 0.80‐1.95).^29^ Similar, the American Society of Anaesthesiologists (ASA) physical status score was not associated with discontinuation in the study by van Erning (2016) (OR_III‐IV vs I‐II_: 0.89; 95% CI: 0.41‐1.94).^39^

#### 3.4.2 Tumor characteristics

Van der Geest et al (2013) found no statistically significant difference in the odds of discontinuation between patients with stage IIIA, IIIB, or IIIC tumors (OR_IIIC vs IIIA/B_: 1.44; 95% CI: 0.89‐2.33).^38^ Similarly, the presence of a mucinous response (OR_present vs absent_: 1.01; 95% CI: 0.65‐1.57) or a lymphocytic response (OR_present vs absent:_ 0.93; 95% CI: 0.56‐1.53) were not associated with discontinuation in the study by Morris et al (2007).^33^ Kumar et al (2015) found that the presence of an obstruction or a perforation was associated with an increased odds of discontinuation (OR: 1.82; 95% CI: 1.08‐3.05).^32^ In contrast, Morris et al (2007) found that the presence of a perforation only was not associated with discontinuation (OR: 0.68; 95% CI: 0.32‐1.43).^33^

#### 3.4.3 Chemotherapy regimen

A quantitative synthesis of the evidence surrounding chemotherapy regimen and the odds of discontinuation was not conducted because of clinical heterogeneity in the study populations and statistical heterogeneity in the effect estimates. Instead, the results from these studies will be qualitatively described. In the MOSAIC Trial (2004), patients randomized to 5‐flourouracil plus oxaliplatin (FOLFOX) had a significantly higher odds of discontinuation than those randomized to 5‐flourouracil alone (OR: 2.16; 95% CI: 1.74‐2.69).^3^ Similarly, van Erning (2016) found that patients aged 70 + years who were prescribed capecitabine plus oxaliplatin (CAPOX) in a real‐world setting had a higher odds of discontinuation relative to those prescribed capecitabine monotherapy (OR: 2.51; 95% CI: 1.63‐3.86).^39^ In contrast, Abrams et al (2011) found that, in a real‐world cancer patient population, combination therapy was associated with a reduced odds of discontinuation relative to monotherapy (OR: 0.78; 95% CI: 0.62‐0.98).^26^ With respect to monotherapies, patients in the X‐ACT Trial (2012) who were randomized to capecitabine monotherapy had a higher odds of discontinuation than those randomized to 5‐flououracil monotherapy (OR: 1.37; 95% CI: 1.07‐1.76).^5^ While CAPOX and FOLFOX have never been compared in a head‐to‐head trial, non‐randomized analyses from the IDEA Trial (2018)^4^ and from Sha (2018)^36^ suggest that the odds of discontinuation is higher among patients who receive CAPOX relative to those who receive FOLFOX (IDEA Trial (2018) 3‐month arm, OR_CAPOX vs FOLFOX_: 1.49; 95% CI: 1.27‐1.75. IDEA Trial (2018) 6‐month arm, OR_CAPOX vs FOLFOX_: 1.30; 95% CI: 1.16‐1.45. Sha (2018), OR_CAPOX vs FOLFOX_: 1.60; 95% CI: 0.91‐2.80).

#### 3.4.4 Provider and other treatment‐related factors

There were reports of a higher odds of discontinuation among patients seen by medical oncologists (OR_6 or fewer patients annually vs 19+ patients_: 1.54; 95% CI: 1.15‐2.04)^26^ and surgeons (OR_low vs high case load_: 2.06; 95% CI: 0.99‐4.25) with lower patient volumes.^33^ Morris et al (2007) found that patients who had not had a preoperative colonoscopy or sigmoidoscopy had a higher odds of discontinuation (OR_no vs yes_: 1.69; 95% CI: 1.13‐2.52).^33^ The urgency of surgery (OR_emergency vselective_: 0.92; 95% CI: 0.42‐2.03),^38^ number of lymph nodes removed (OR_≥12 vs <12 nodes_: 1.33; 95% CI: 0.87‐2.04),^32^ re‐operation (OR_yes vs no:_ 1.48; 95% CI: 0.61‐3.59),^38^ and time to chemotherapy initiation (OR_>8 vs_ _<8__ weeks_: 1.16; 95% CI: 0.81‐1.68)^32^ were not associated with discontinuation among the studies that examined these factors. Yoshida et al (2015) found that the type of surgical procedure was associated with discontinuation (OR_colectomy vs low anterior or abdominal resection_: 2.00; 95% CI: 1.15‐2.45)^40^ whereas van der Geest et al (2013) found that the mode of surgical access (open vs laparoscopic) was not associated with discontinuation (OR:_open vs laparoscopic_: 1.40; 0.87‐2.27).^38^

### 3.5 Risk of bias assessment

Since all of the studies included in this review were based on administrative, registry, and medical abstract data and because of the short duration of follow‐up needed to assess chemotherapy discontinuation, the risk of bias in the “study population”, “study attrition”, and “outcome measurement” domains of the QUIP tool were judged to be low within all studies (Table 4). Three of the 18 studies were assigned a moderate risk of bias in the “prognostic factor measurement” domain because they dichotomized one or more continuous variables. Regarding the “adjustment for other prognostic factors” domain, nine studies were deemed to have a high risk of bias. Lastly, three studies were judged to have a moderate risk of bias in the “statistical analysis and reporting” domain as the reporting of outcomes appeared to be guided by a *P*‐value.

**Table 4:** Quality in Prognostic Factor Studies (QUIPS) risk of bias assessment^20^ among studies included in the systematic review (n = 18)

Study	1. Study participation	2. Study attrition	3. Prognostic factor measurement	4. Outcome measurement	5. Adjustment for other prognostic factors (No. of Variables in Model)^a^	6. Statistical analysis and reporting
Observational studies						
Abrams (2011)^26^	Low	Low	Low	Low	Low (8)	Low
Brungs (2018)^27^	Low	Low	Moderate	Low	High (1)	Low
Cespedes Feliciano (2017)^28^	Low	Low	Low	Low	Moderate (3)	Low
Hu (2011)^29^	Low	Low	Low	Low	Low (11)	Low
Jensen (2006)^30^	Low	Low	Moderate	Low	High (1)	Low
Kahn (2010)^31^	Low	Low	Low	Low	High (1)	Low
Kumar (2015)^32^	Low	Low	Moderate	Low	Low (14)	Low
Morris (2007)^33^	Low	Low	Low	Low	High/Low (1/16)^b^	Moderate
Romanus (2009)^34^	Low	Low	Low	Low	Moderate (3)	Low
Sgouros (2015)^35^	Low	Low	Low	Low	High (1)	Low
Sha (2018)^36^	Low	Low	Low	Low	High (1)	Low
Sun (2015)^37^	Low	Low	Low	Low	High (1)	Low
van der Geest (2013)^38^	Low	Low	Low	Low	High/Moderate (1/3)^b^	Moderate
van Erning (2016)^39^	Low	Low	Low	Low	Low (10)	Low
Randomized clinical trials						
IDEA Trial (2018)^4^	Low	Low	Low	Low	High (1)	Low
JCOG0910 Trial (2015)^40^	Low	Low	Low	Low	Low (9)	Moderate
MOSAIC Trial (2004)^3^	Low	Low	Low	Low	Low (Not Necessary)	Low
X‐ACT Trial (2012)^5^	Low	Low	Low	Low	Low (Not Necessary)	Low

a Some studies did not include all measured covariates within the multivariable model. As such, the number of prognostic factors examined within the study (as reported in Table 2) may not correspond with the number of variables within the multivariable model (as reported in Table 4).

b Multivariable adjusted estimates were available for some variables whereas only crude estimates were available for others (see Table S1)

### 3.6 Publication bias

There was no evidence of publication bias among the age‐specific results based on an examination of a funnel plot (Figure S18). An assessment of publication bias was not conducted for any other variables because the number of studies was less than 10.^13^

## 4 DISCUSSION

The evidence to date suggests that as many as 2 in 3 patients will discontinue adjuvant chemotherapy in real‐world settings depending upon the outcome definition and study population. The primary objective of this study was to identify factors that are prognostic of adjuvant chemotherapy discontinuation among stage III colon cancer patients. With respect to the survey of medical oncologists, firm conclusions cannot be made given the lack of agreement across clinicians which suggests that medical oncologists may be unable to accurately determine which patients have a high risk of treatment discontinuation. Nonetheless, there was some suggestion that clinicians thought that age, comorbidity, time to chemotherapy initiation, baseline laboratory values, and tumor stage were important prognostic factors for chemotherapy discontinuation in this population. Results from the systematic review and meta‐analysis suggested that comorbidity, chemotherapy regimen, performance status, t stage, and tumor grade were significantly associated with chemotherapy discontinuation. Summarizing across these bodies of evidence, the following variables were identified as having strong evidence of being prognostic of early discontinuation because they were supported by both the opinions of medical oncologists and by findings from multiple investigations: (a) comorbidity; (b) t stage; (c) performance status; and (d) chemotherapy regimen (Table 5). With respect to chemotherapy regimen, the literature suggests that CAPOX has the highest odds of discontinuation followed by FOLFOX, capecitabine monotherapy, and 5‐flououracil monotherapy. The finding from Abrams et al (2011) that patients prescribed a combination therapy had a lower odds of discontinuation relative to those prescribed a monotherapy is noteworthy.^26^ This finding suggests that receipt of a monotherapy may be a proxy of poor candidacy for oxaliplatin and that patients who are poor candidates for oxaliplatin have a higher odds of discontinuing chemotherapy.

**Table 5:** Prognostic Variables Identified from this Investigation and Source of Evidence

Variable	Source of supporting evidence		
	Clinician survey	Meta‐analysis	Narrative summary
Strong evidence			
Chemotherapy Regimen	X^a^		X
Comorbidity	X	X	
Performance Status	X^a^	X	
T Stage	X	X	
Some evidence			
Age	X	Some support	
Marital Status (Social Support)	X^a^		X
Muscle Mass	X^a^		X
N stage	X	Some support	
Preoperative colonoscopy or sigmoidoscopy			X
Tumor Grade		X	
Insufficient evidence			
Clinician volume			X
Ethnicity			X
Laboratory measures	X		
Presence of an Obstruction			X
Prolonged hospital stay duration		Some support	
Gender		Some support	
Time to chemotherapy initiation	X		
Treatment facility		Some support	
Type of surgical procedure			X

a Not originally included in survey but identified by one or more clinicians as having prognostic importance

### 4.1 Limitations

There are limitations of this investigation that should be acknowledged. With respect to our survey of medical oncologists, the absolute number of participants was small. In addition, our survey targeted clinicians who treated colon cancer within Alberta, Canada. For these reasons, our findings may not be generalizable, particularly to medical oncologists who work within other geographic regions and who treat other types of cancer. In addition, our method of expert elicitation was simplistic in that the interpretation of variable importance likely differed between clinicians which could account for the low degree of agreement. For many of the variables examined in our systematic review and meta‐analysis, the number of investigations was small, the estimates were imprecise, and/or the degree of heterogeneity was substantial. Therefore, the strength of the conclusions that can be drawn from these findings is limited. Similarly, the number of participants included in the analyses was small for a number of the comparisons within this review, particularly those describe within the narrative summary. The lack of statistical significance that was observed for some of these comparisons may therefore be attributable to a lack of statistical power arising from limited sample sizes. Also, our study population of interest was stage III colon cancer patients. As such, these findings are not generalizable to individuals with metastatic disease or with malignancies in other sites. Lastly, the studies included in this review were limited with respect to the scope of the covariates that were examined as they relied primarily on routinely collected clinical data. As such, there are likely important prognostic factors that have yet to be quantitatively examined.

### 4.2 Areas for future research

There is a need for additional research to identify factors that are prognostic of early discontinuation of adjuvant chemotherapy among stage III colon cancer patients. New investigations should target variables that we identified as being prognostic or potentially prognostic to confirm the findings from our systematic review and meta‐analysis. In addition to these variables, researchers should focus on the variables that medical oncologists considered to be potentially important that were not examined within any of the studies included in our systematic review. These variables included patient‐level factors such as mental health, interest in alternative medicines, degree of social support, and health care delivery factors such as distance from home to treatment facility. Additional qualitative research and large‐scale population‐based surveys of patients and clinicians may help to expand this list by identifying additional prognostic factors that have yet to be examined within the quantitative literature. In addition, many of the studies in this review relied upon retrospective administrative data and, as a result, were unable to assess easily measured lifestyle factors such as a history of tobacco or alcohol use which could be explored in future analyses. Several randomized trials were excluded because they did not provide a comparison between a prognostic factor and chemotherapy discontinuation or because they compared the risk of discontinuation relative to a treatment not used within clinical practice (eg FOLFOX versus FOLFOX + bevacizumab or FOLFOX + cetuximab).^41^, ^42^ In addition, the MOSIAC Trial, X‐ACT Trial, and IDEA Trial were included in this review solely because we were able to indirectly estimate an association between treatment regimen and discontinuation using reported data.^3^, ^4^, ^5^ We therefore highlight the existence of a large amount of clinical trial data that could be used to further help identify determinants of chemotherapy discontinuation. Researchers could leverage these data by conducting multivariable‐adjusted analyses that are restricted to patients who received a regimen currently approved for use within this patient population. While many of the studies included in this review used a multivariable model, no investigation to date assessed the performance of a multivariable model as a clinical prediction tool. Moreover, it would be difficult to assess the external validity of existing multivariable models because the regression coefficient corresponding to the intercept was not reported. Future research should report the intercept from multivariable models and could focus on the development of clinician decision support tools for predicting early chemotherapy discontinuation. It is noteworthy that only one study included within this review examined cancer stage according to the overall TNM stage variable and that all other studies modeled T and N stage as separate covariates in which additivity was assumed. Future researchers should consider using the overall TNM stage variable since the number of parameters needed to fully specify all possible T1‐4 and N1‐2 strata would be seven whereas only two parameters are needed to fully specify strata defined by TNM stage IIIA‐C. Additionally, there is a great deal of heterogeneity in the literature regarding the operationalization of chemotherapy discontinuation and there is a need to reach consensus regarding the definition that should be used in future investigations. Lastly, it should be noted that none of the investigations stratified the analyses by reason for discontinuation. Such stratification may provide additional insight regarding the prognostic value of the variables examined.

## 5 CONCLUSIONS

While the literature is heterogeneous and limited, there is evidence that comorbidity, chemotherapy regimen, performance status, and T stage are prognostic factors for early discontinuation of chemotherapy. Further research is needed to confirm these findings, to assess additional variables that have yet to be examined, to develop clinical prediction tools, and to reach consensus regarding the definition of chemotherapy discontinuation that should be used within this body of literature.

## 6 AUTHOR CONTRIBUTIONS STATEMENT

Devon J Boyne: Conceptualization, data curation, formal analysis, methodology, writing‐original draft, and writing‐review and editing. Dylan E O'Sullivan: Data curation, methodology, writing‐review and editing. Emily V Heer: Data curation, writing‐review and editing. Robert J Hilsden, Tolulope T Sajobi, and Darren R Brenner: Conceptualization, supervision, methodology, writing – review and editing. Winson Y Cheung and Christine M Friedenreich: Data curation, conceptualization, supervision, methodology, writing – review and editing.

## CONFLICTS OF INTEREST

The authors declare no conflicts of interest.

## PRECIS

Results from a meta‐analysis of 18 studies and a survey of 14 medical oncologists suggests that comorbidity, performance status, T stage, and chemotherapy regimen are factors that are prognostic of early chemotherapy discontinuation in this population.

## Data Availability

The data that support the findings of this study are available from the corresponding author upon reasonable request. The study‐specific estimates used in the meta‐analysis are available in forest plots in the supplemental file.
