# Supplementary material for: Prognostic factors of adjuvant chemotherapy discontinuation among stage III colon cancer patients: A survey of medical oncologists and a systematic review and meta‐analysis
Source: Cancer Med. 2020 Jan 21;9(5):1613–27. doi: 10.1002/cam4.2843 (PMC7050079; doi:10.1002/cam4.2843)

Table S1. Additional details regarding the type of analysis and multivariable-adjustment within the studies that examined the association between a prognostic factor and chemotherapy discontinuation among patients with stage III colon cancer (n=18)

| **Study** | **Covariates with Missing Data, n missing (%)** | **Type of Analysis** | **No. of Covariates Examined** | **Covariates Examined** | **No. of Covariates in MVA** | **Covariates in MVA** | **Variable Selection**  **Method** |
| --- | --- | --- | --- | --- | --- | --- | --- |
| Abrams (2011)^26^ | ECOG: 550/2501 (22%) | Multivariable logistic regression | 7 | Age, ECOG, oncologist case volume, sex, treatment facility, tumor stage, and type of chemotherapy | 8 | Age, ECOG, oncologist case volume, sex, treatment facility, tumor stage, and type of chemotherapy [additionally adjusted for geographic region] | None |
| Brungs (2018)^27^ | None | Crude proportions | 1 | Age | - | - | - |
| Cespedes Feliciano (2017)^28^ | None | Multivariable logistic regression | 1 | Muscle mass | 4 | Muscle mass, age, sex, disease stage | Expert opinion |
| Hu (2011)^29^ | Grade: 203/4660 (4%)  Race: <10%^b^  Marital status: <5%^b^ | Multivariable logistic regression | 9 | Age, comorbidity, marital status, N stage, race, sex, SES, tumor grade, urban/rural | 11 | Age, comorbidity, geographic region, marital status, N stage, period of diagnosis, race, sex, SES, tumor grade, urban/rural [additionally adjusted for period of diagnosis and geographic region] | None |
| Jensen (2006)^30^ | None | Crude proportions | 1 | Age | - | - | - |
| Kahn (2010)^31^ | None | Crude proportions | 1 | Age | - | - | - |
| Kumar (2015)^32^ | Grade: 9/616 (1.5%) | Multivariable logistic regression | 14 | Age, comorbidity, ECOG, lymphovascular invasion, N stage, nodes removed, obstruction/perforation, perineural invasion, postoperative stay, T stage, tumor grade, tumor side, time to chemotherapy, sex | 14 | Age, comorbidity, ECOG, lymphovascular invasion, N stage, nodes removed, obstruction/perforation, perineural invasion, postoperative stay, T stage, tumor grade, tumor side, time to chemotherapy, sex | None |
| Morris (2007)^33^ | Not reported | Multivariable logistic regression | 15 | Age, lymphocytic response, lymphovascular invasion, perineural invasion, preoperative colonoscopy or sigmoidoscopy, mucinous, N stage, obstruction/perforation, SES, sex, surgical case volume, T stage, treatment facility, tumor grade, tumor side | 16 | Age, lymphocytic response, lymphovascular invasion, perineural invasion, preoperative colonoscopy or sigmoidoscopy, mucinous, N stage, obstruction/perforation, SES, sex, surgical case volume, T stage, treatment facility, tumor grade, tumor side [additionally adjusted for period of diagnosis]  NOTE: multivariable adjusted estimates were only available for age, sex, site, surgical case volume, SES, and treatment facility | None |
| Romanus (2009)^34^ | None | Multivariable logistic regression | 4 | Age, history of diabetes, tumor stage | 5 | Age, history of diabetes, tumor stage [additionally adjusted for treatment centre] | None |
| Sgouros (2015)^35^ | None | Crude proportions | 1 | Tumor stage | - | - | - |
| Sha (2018)^36^ | None | Crude proportions | 1 | Type of chemotherapy | - | - | - |
| Sun (2015)^37^ | None | Crude proportions | 4 | Age^a^, ECOG, sex, tumor stage | - | - | - |
| van der Geest (2013)^38^ | None | Multivariable logistic regression | 10 | Age, comorbidity, prolonged hospital stay, reoperation, SES, sex, surgical procedure, tumor grade, tumor side, tumor stage, urgency of surgery | 3 | Age, prolonged hospital stay, sex | Backwards Elimination (p-value < 0.10) |
| van Erning (2016)^39^ | Comorbidity: 5/193 (2.6%)  ASA score: 39/193 (20.2%)  Tumor side: <2%^b^  Tumor grade: 11/193 (5.7%) | Multivariable logistic regression | 9 | Age, ASA score, comorbidity, N stage, period of diagnosis sex, T stage, tumor grade, tumor side, type of chemotherapy | 10 | Age, ASA score, comorbidity, N stage, period of diagnosis sex, T stage, tumor grade, tumor side, type of chemotherapy [additionally adjusted for period of diagnosis and treatment toxicity] | None |
| IDEA Trial (2018)^4^ | None | Crude Proportions | 1 | Type of chemotherapy  (not randomized) | - | - | - |
| JCOG0910 Trial (2015)^40^ | Excluded 8/774 (1.0%) patients from initial cohort due to missing data | Multivariable logistic regression | 2 | Age, surgical procedure | 10 | Age, sex, body surface area, body mass index, surgical procedure, creatinine clearance rate, aspartate aminotransferase, alanine aminotransferase, total bilirubin, treatment toxicity | None |
| MOSAIC Trial (2004)^3^ | None | Crude Proportions | 1 | Type of chemotherapy | - | - | - |
| X-ACT Trial (2012)^5^ | None | Crude Proportions | 1 | Type of chemotherapy | - | - | - |

Abbreviations: MVA = multivariable analysis

^a^ Indicator for whether or not one of the study’s objectives was to identify predictors of chemotherapy discontinuation

^b^ Individuals with missing data were grouped with individuals who were classified as “other”. As such, we are unable to tell the proportion of patients who were missing data on this covariate.

Figure S1. Meta-Analysis of the Association between Age and Chemotherapy Discontinuation among Stage II/III Colon Cancer Patients


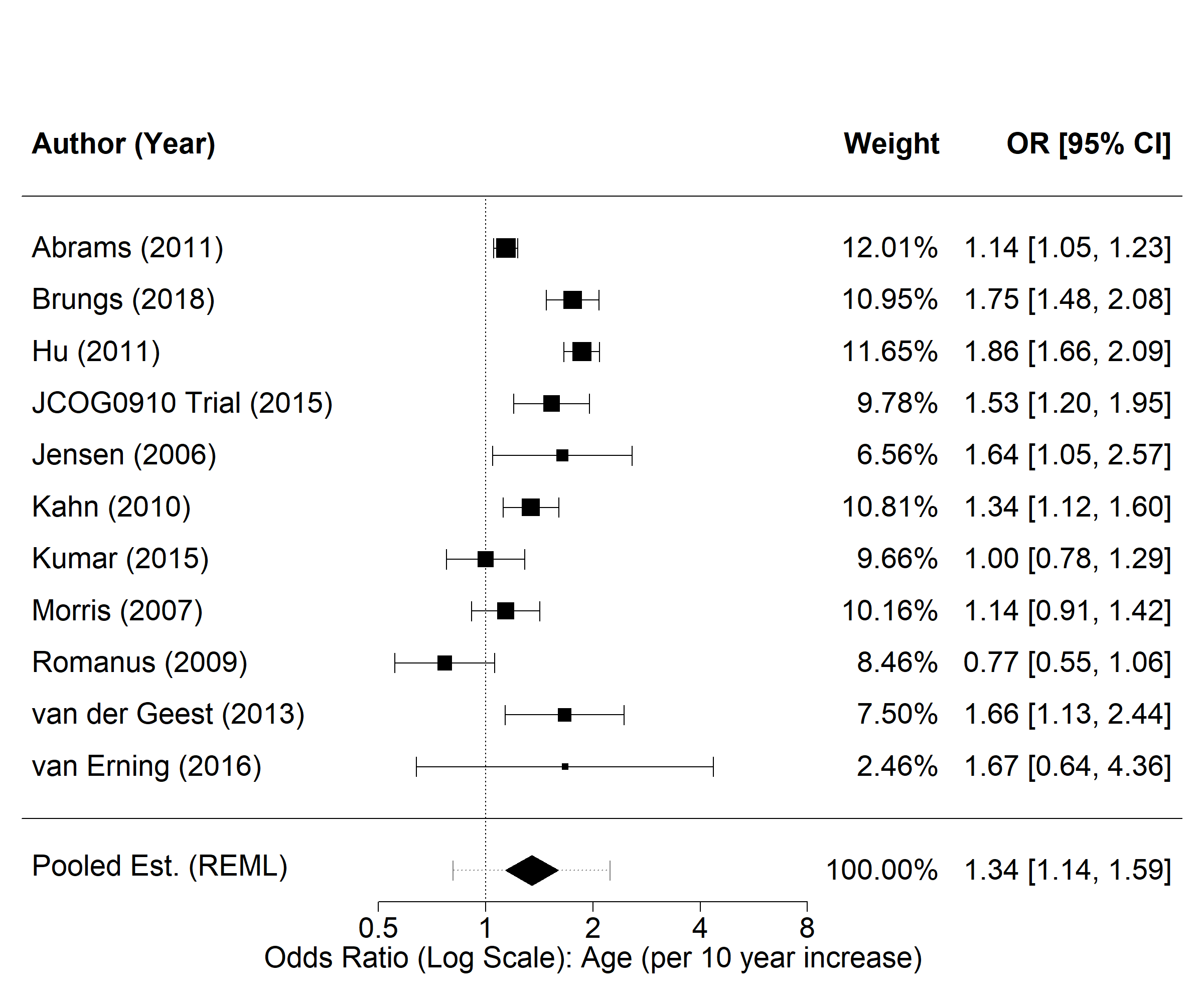


Figure S2. Meta-Analysis of the Association between Sex and Chemotherapy Discontinuation among Stage II/III Colon Cancer Patients


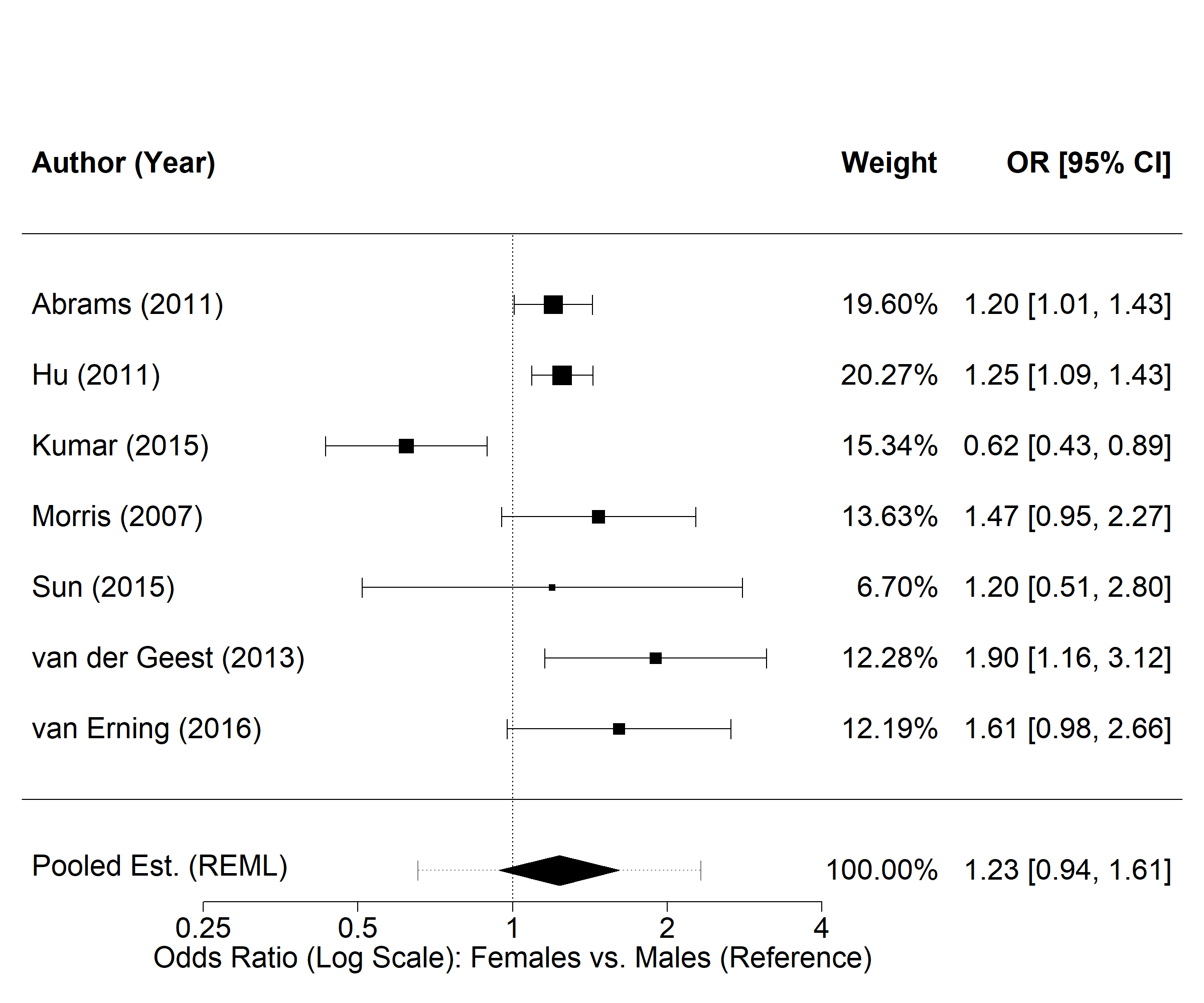


Figure S3. Meta-Analysis of the Association between Socioeconomic Status and Chemotherapy Discontinuation among Stage II/III Colon Cancer Patients


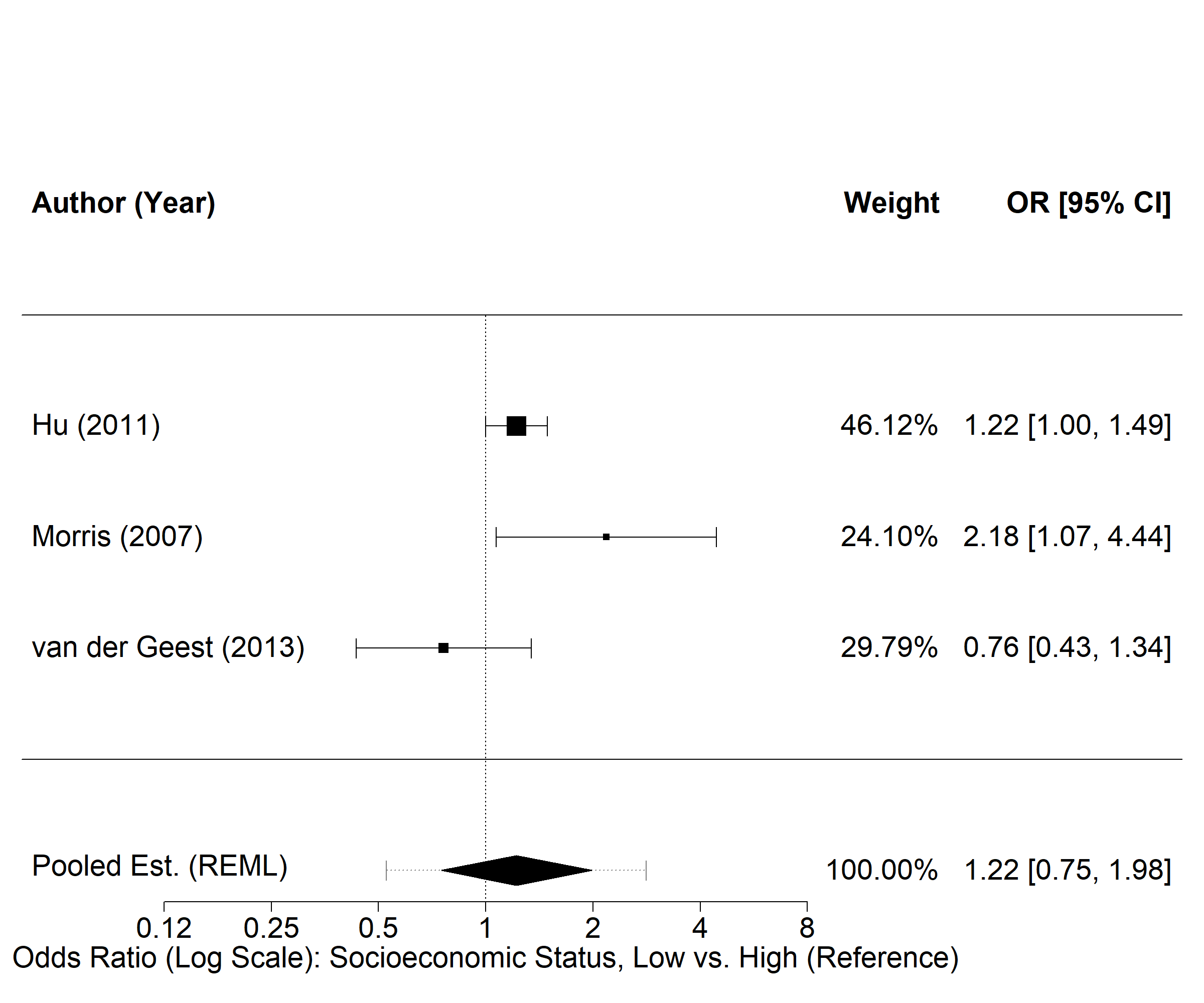


Figure S4. Meta-Analysis of the Association between Comorbidity (2+ vs. 0) and Chemotherapy Discontinuation among Stage II/III Colon Cancer Patients


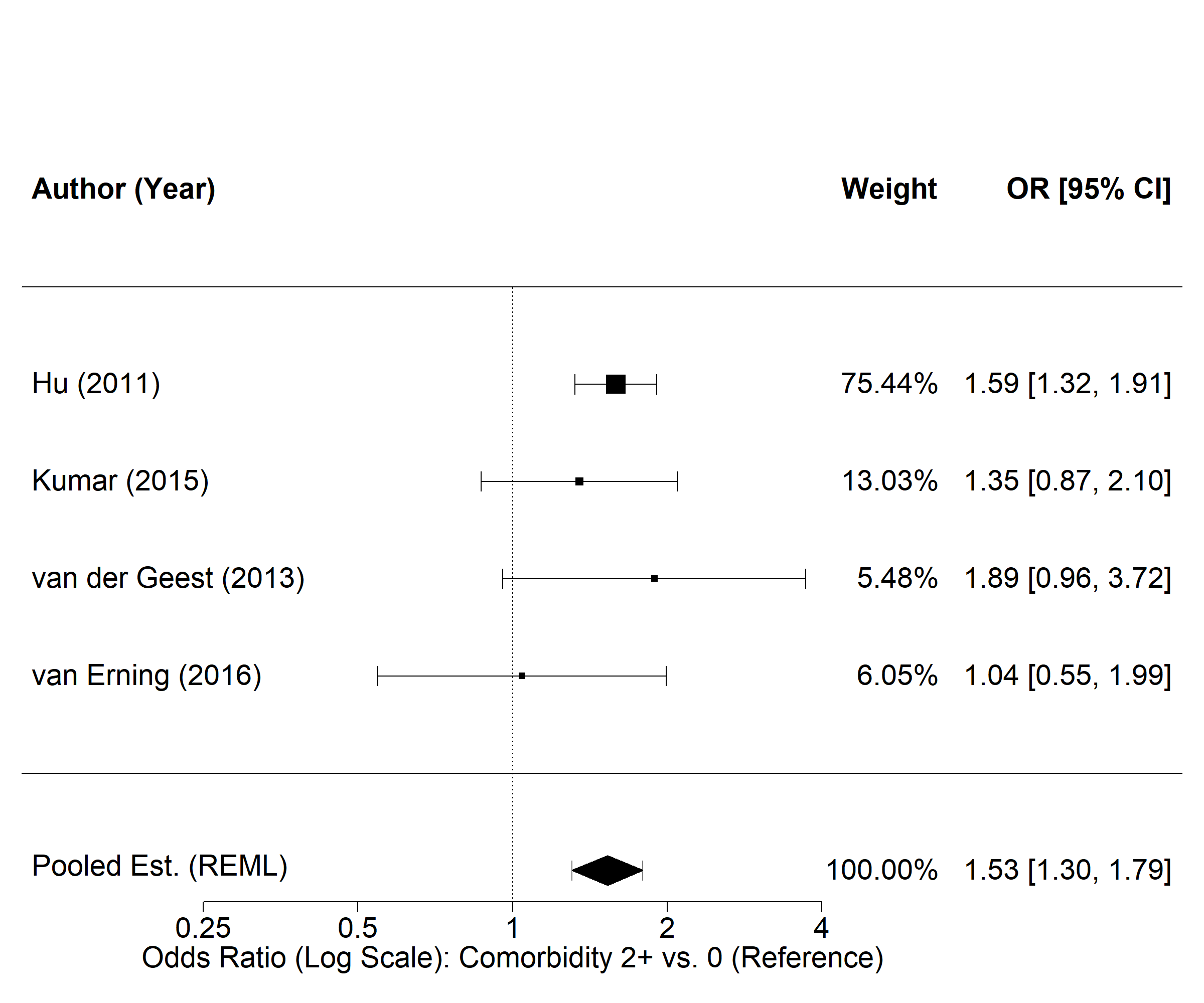


Figure S5. Meta-Analysis of the Association between Comorbidity (1 vs. 0) and Chemotherapy Discontinuation among Stage II/III Colon Cancer Patients


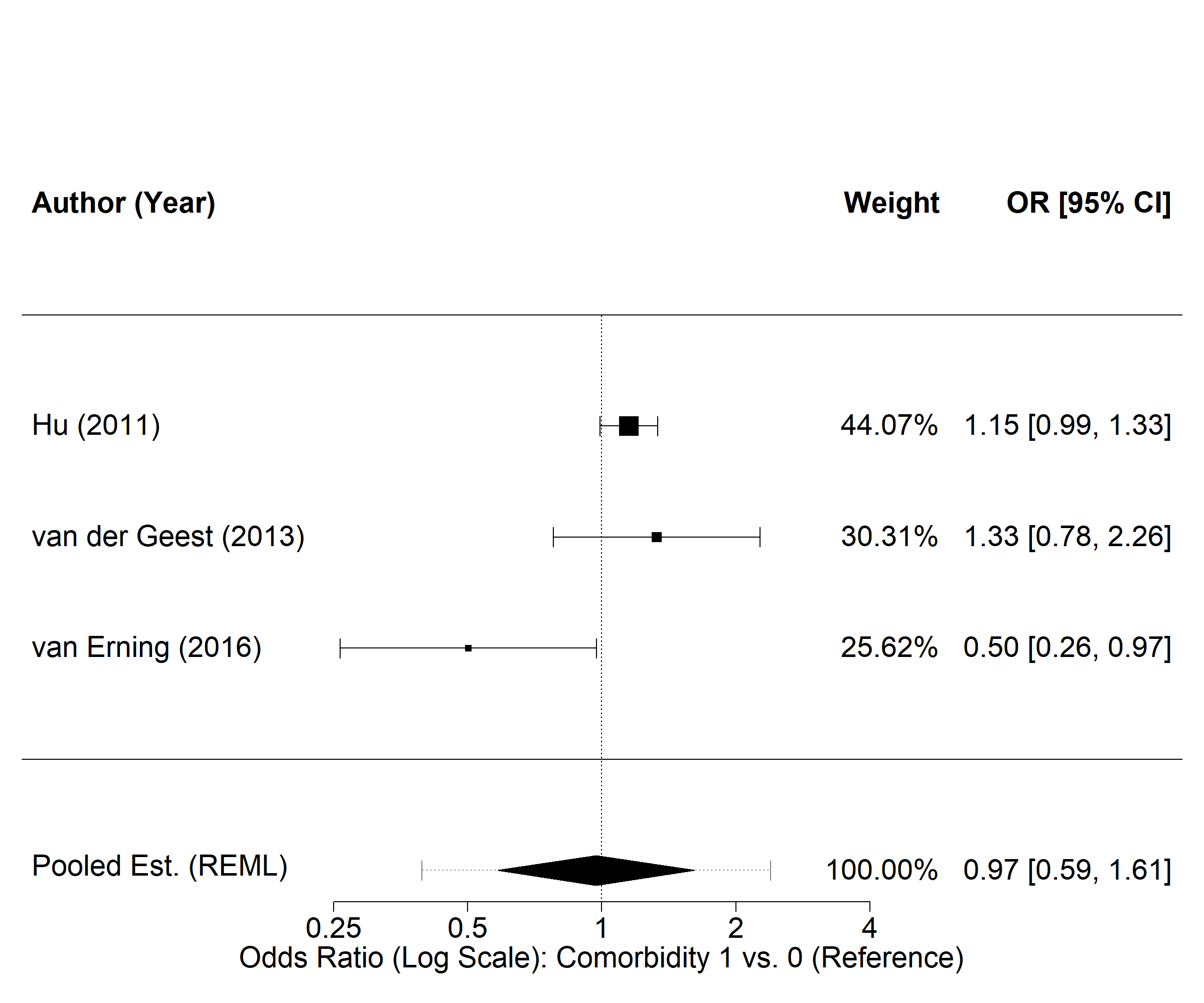


Figure S6. Meta-Analysis of the Association between ECOG Score and Chemotherapy Discontinuation among Stage II/III Colon Cancer Patients


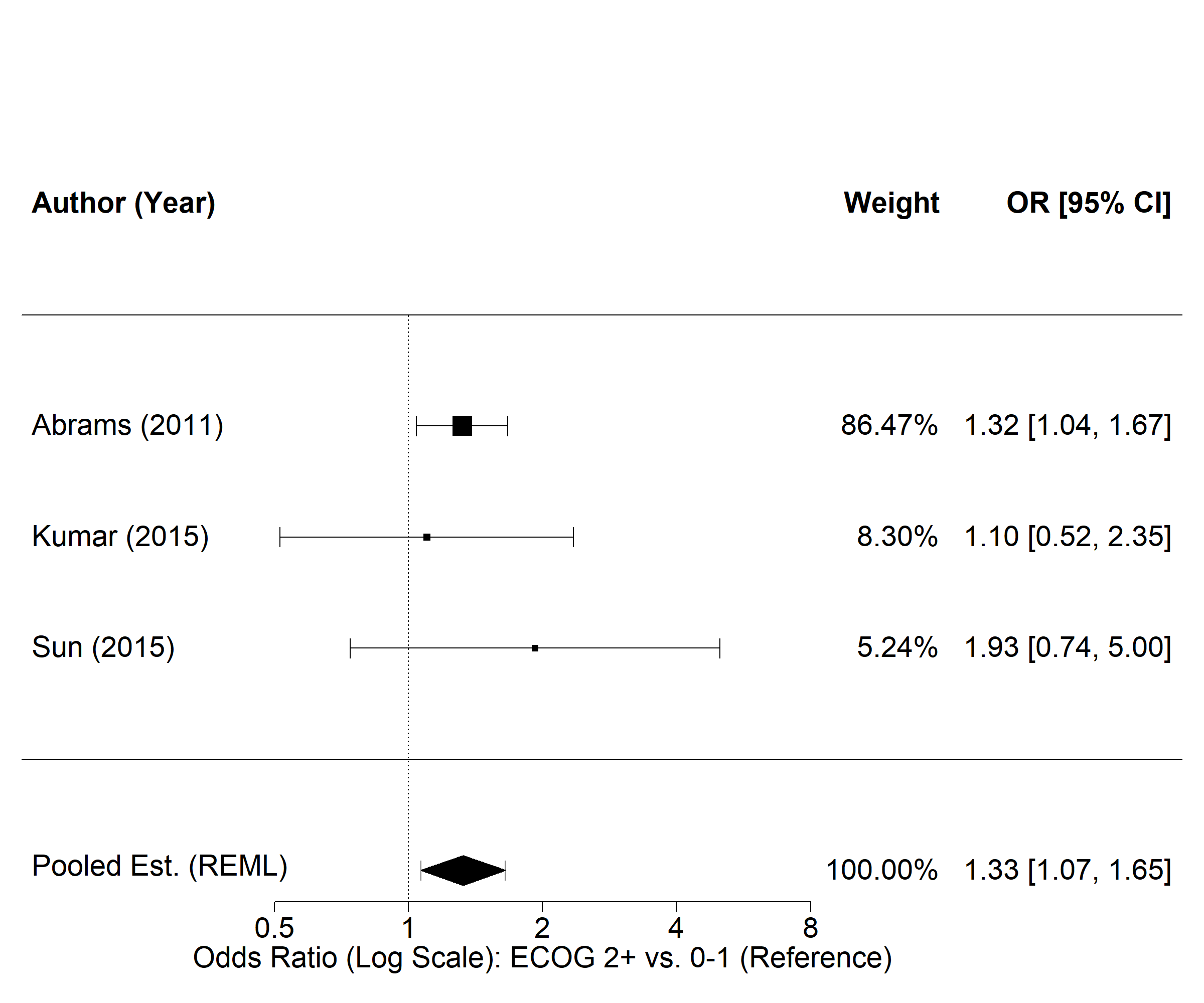


Figure S7. Meta-Analysis of the Association between Tumor Stage and Chemotherapy Discontinuation among Stage II/III Colon Cancer Patients


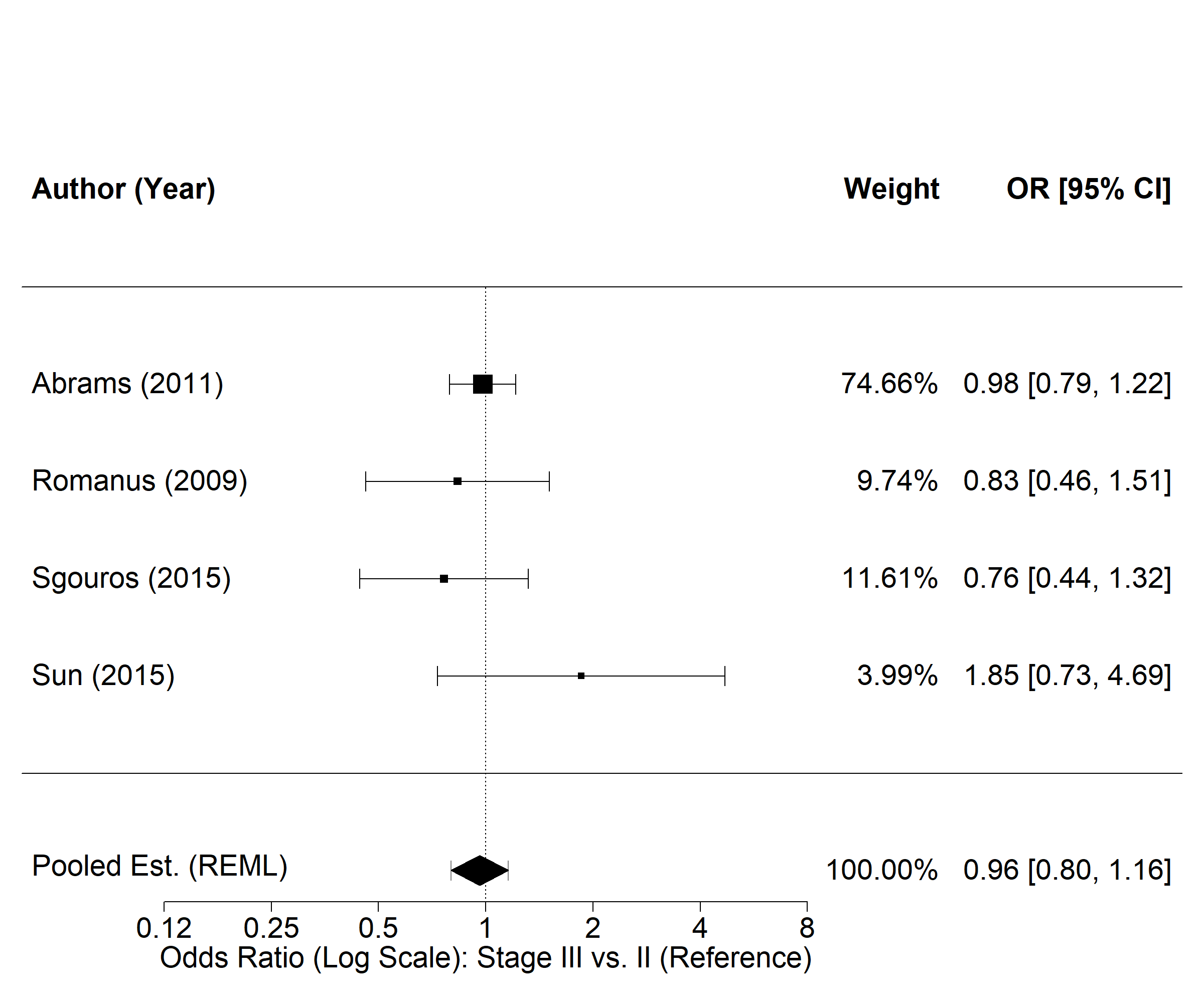


Figure S8. Meta-Analysis of the Association between T Stage (T4 vs. T1-2) and Chemotherapy Discontinuation among Stage II/III Colon Cancer Patients


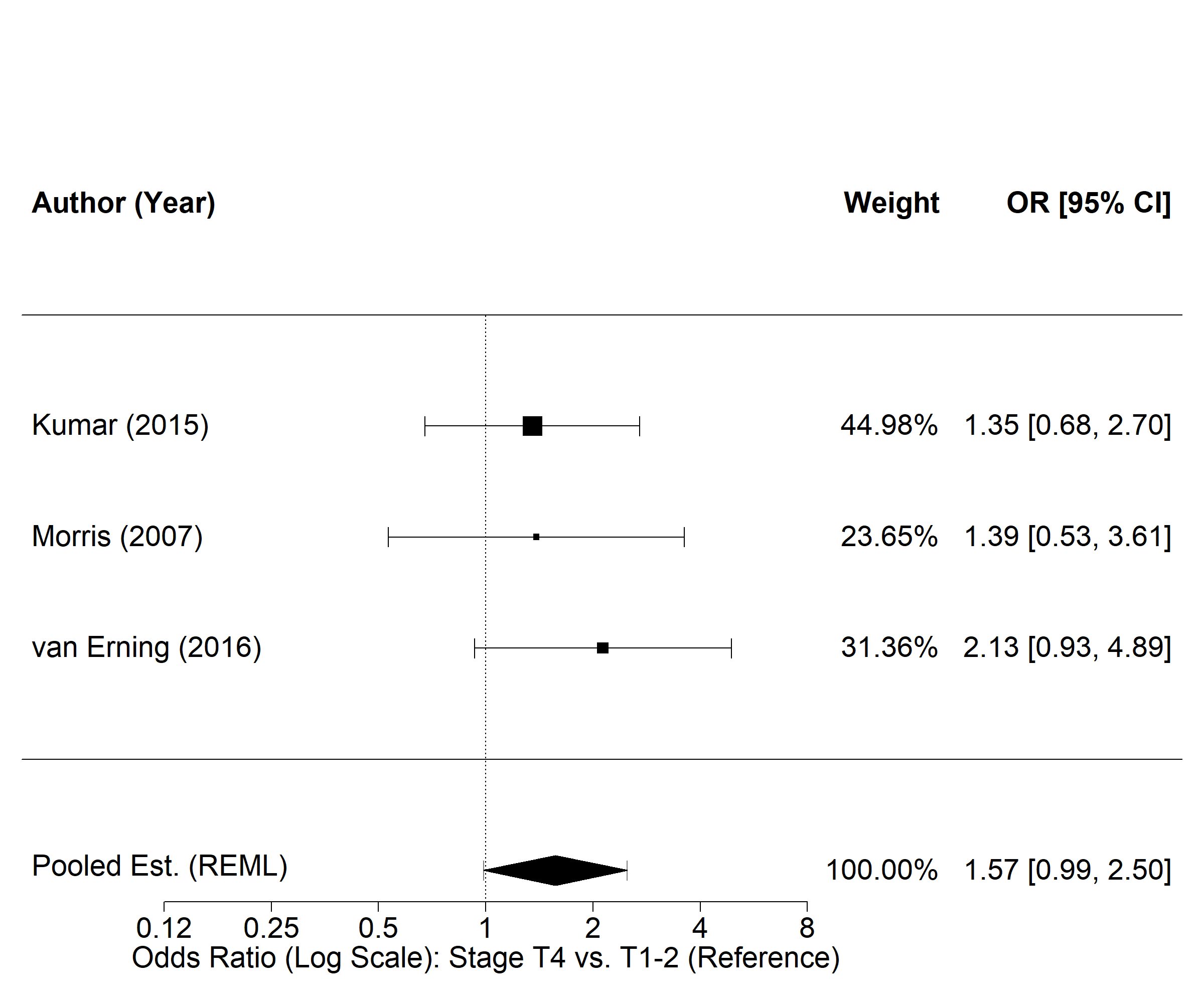


Figure S9. Meta-Analysis of the Association between T Stage (T3 vs. T1-2) and Chemotherapy Discontinuation among Stage II/III Colon Cancer Patients


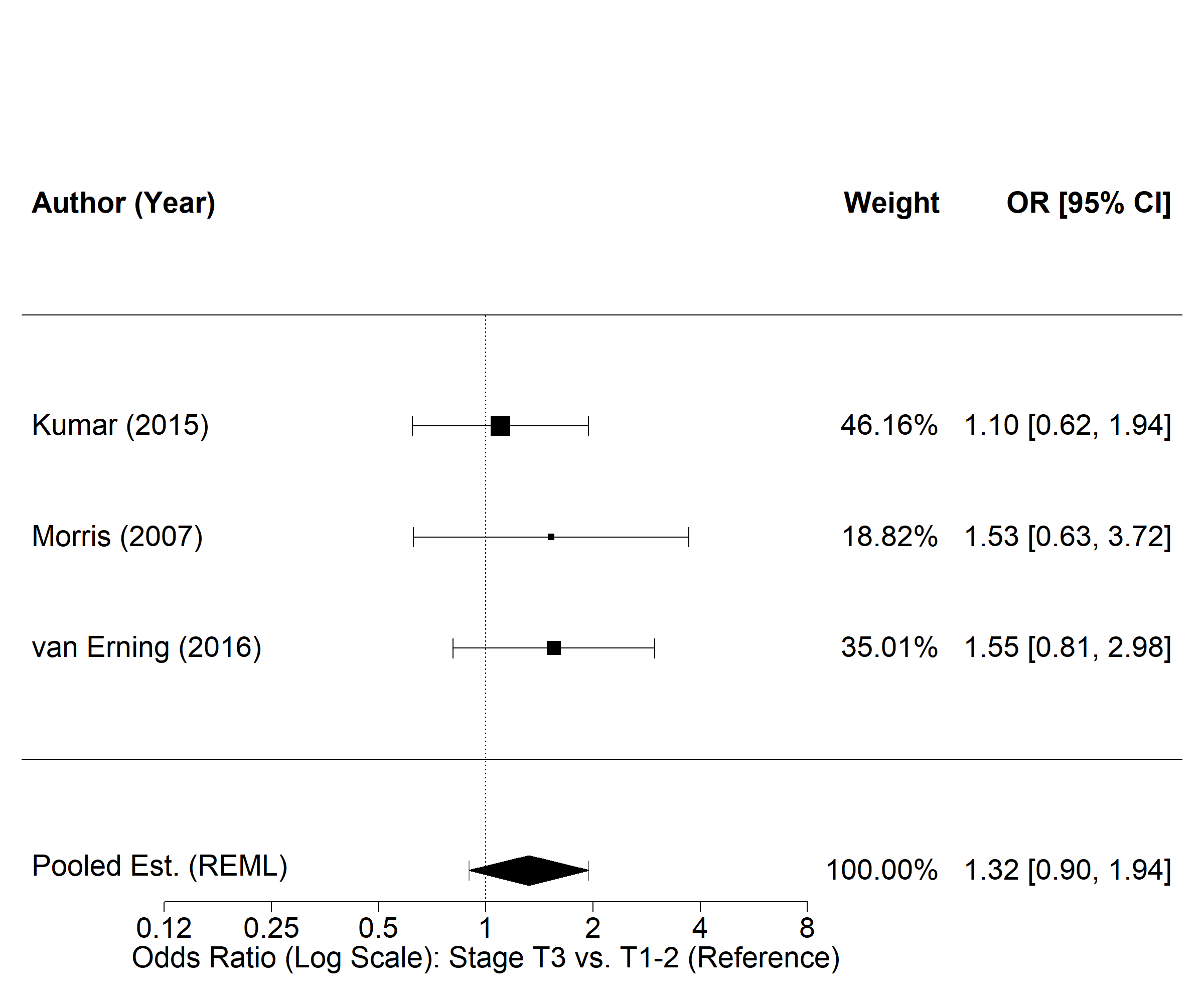


Figure S10. Meta-Analysis of the Association between N Stage and Chemotherapy Discontinuation among Stage II/III Colon Cancer Patients


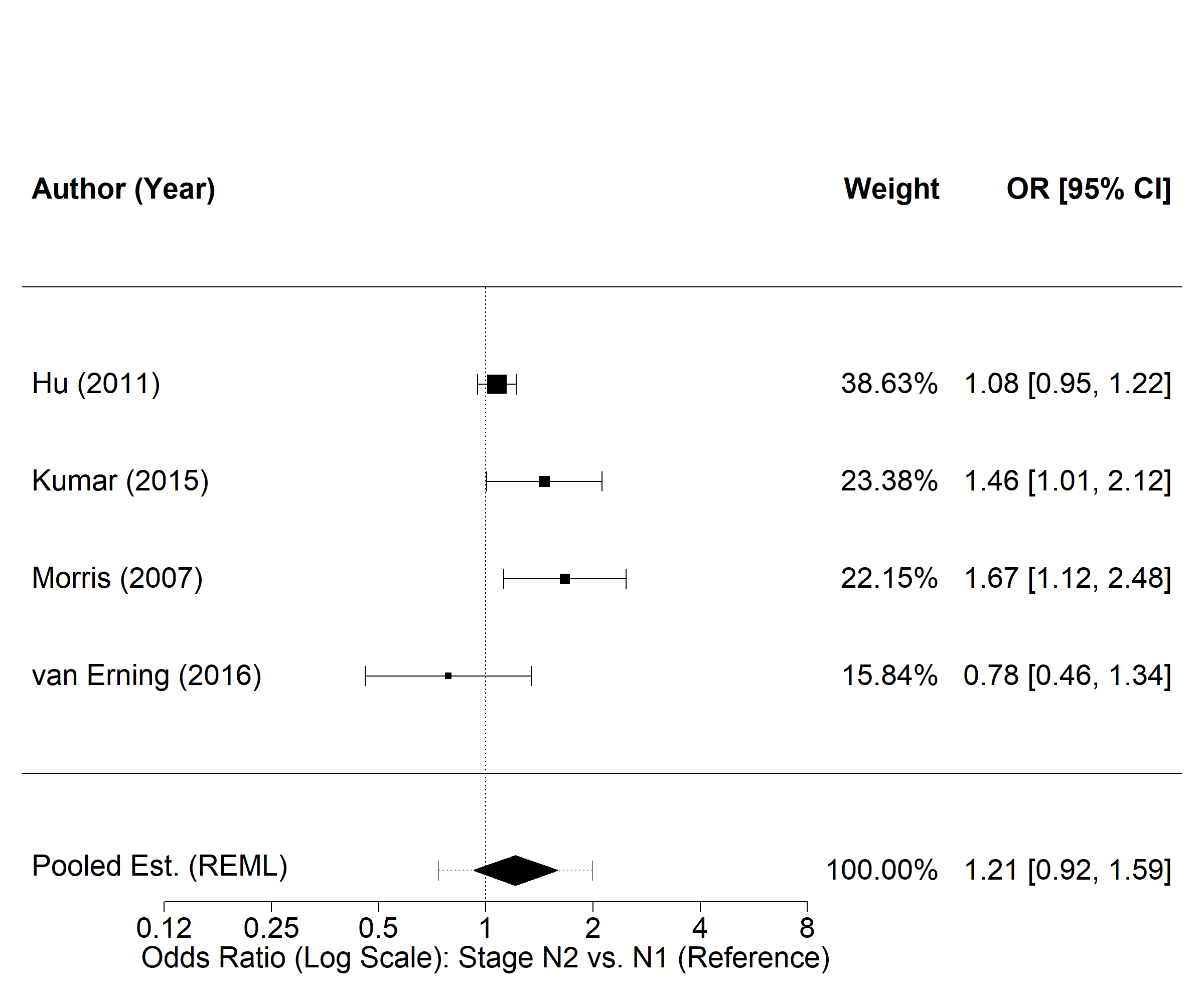


Figure S11. Meta-Analysis of the Association between Tumor Side and Chemotherapy Discontinuation among Stage II/III Colon Cancer Patients


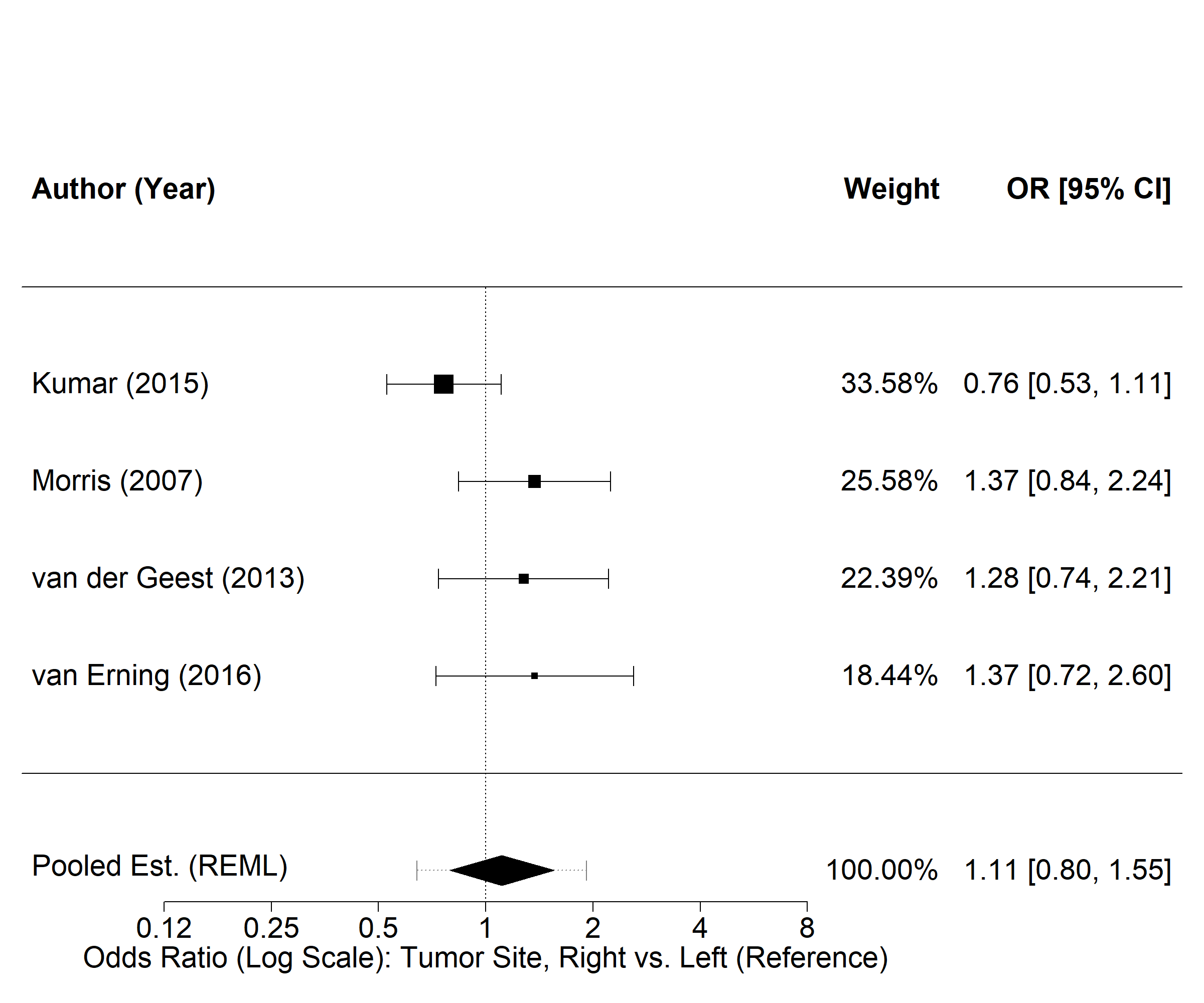


Figure S12. Meta-Analysis of the Association between Tumor Grade and Chemotherapy Discontinuation among Stage II/III Colon Cancer Patients


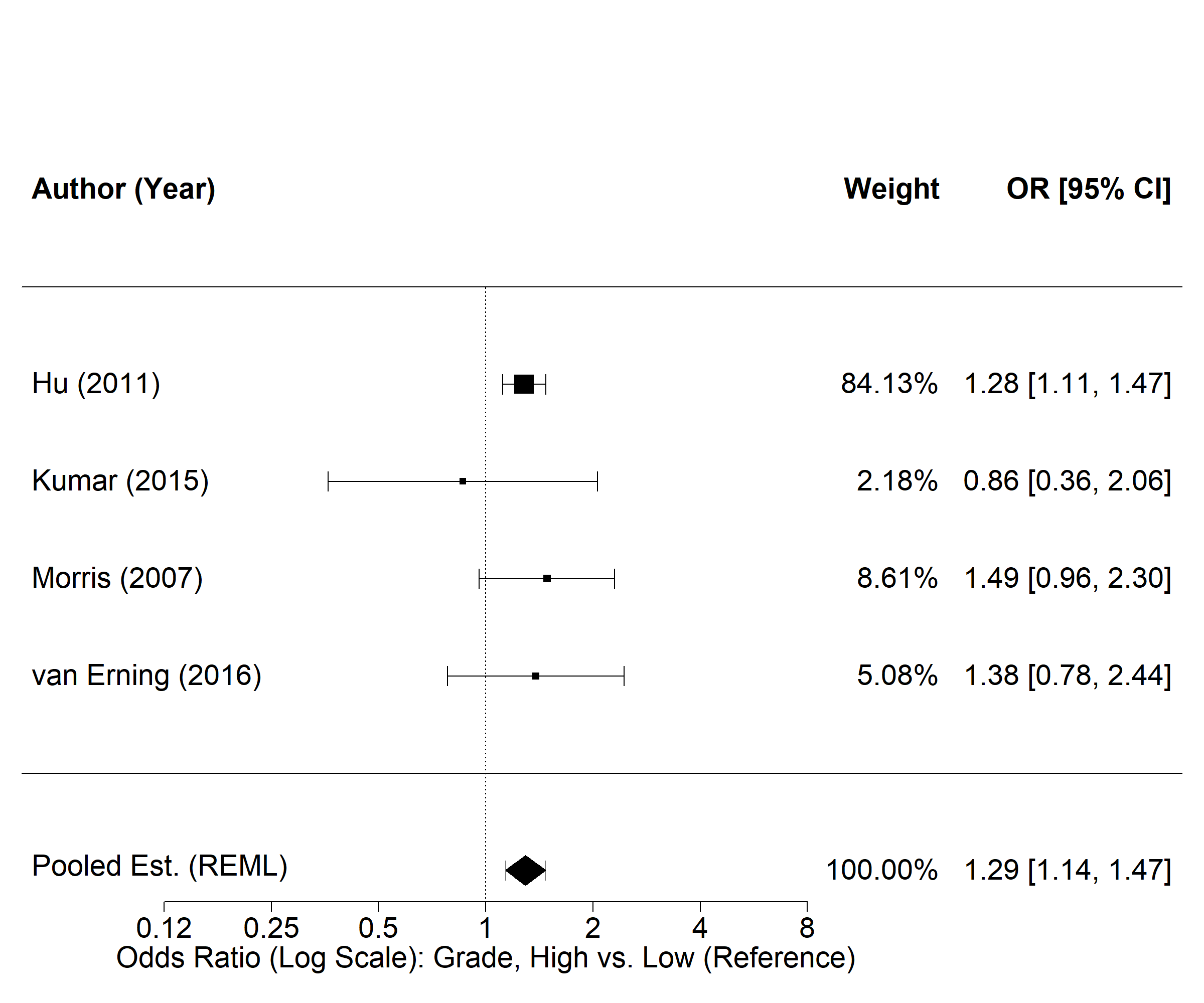


Figure S13. Meta-Analysis of the Association between Lymphovascular Invasion and Chemotherapy Discontinuation among Stage II/III Colon Cancer Patients


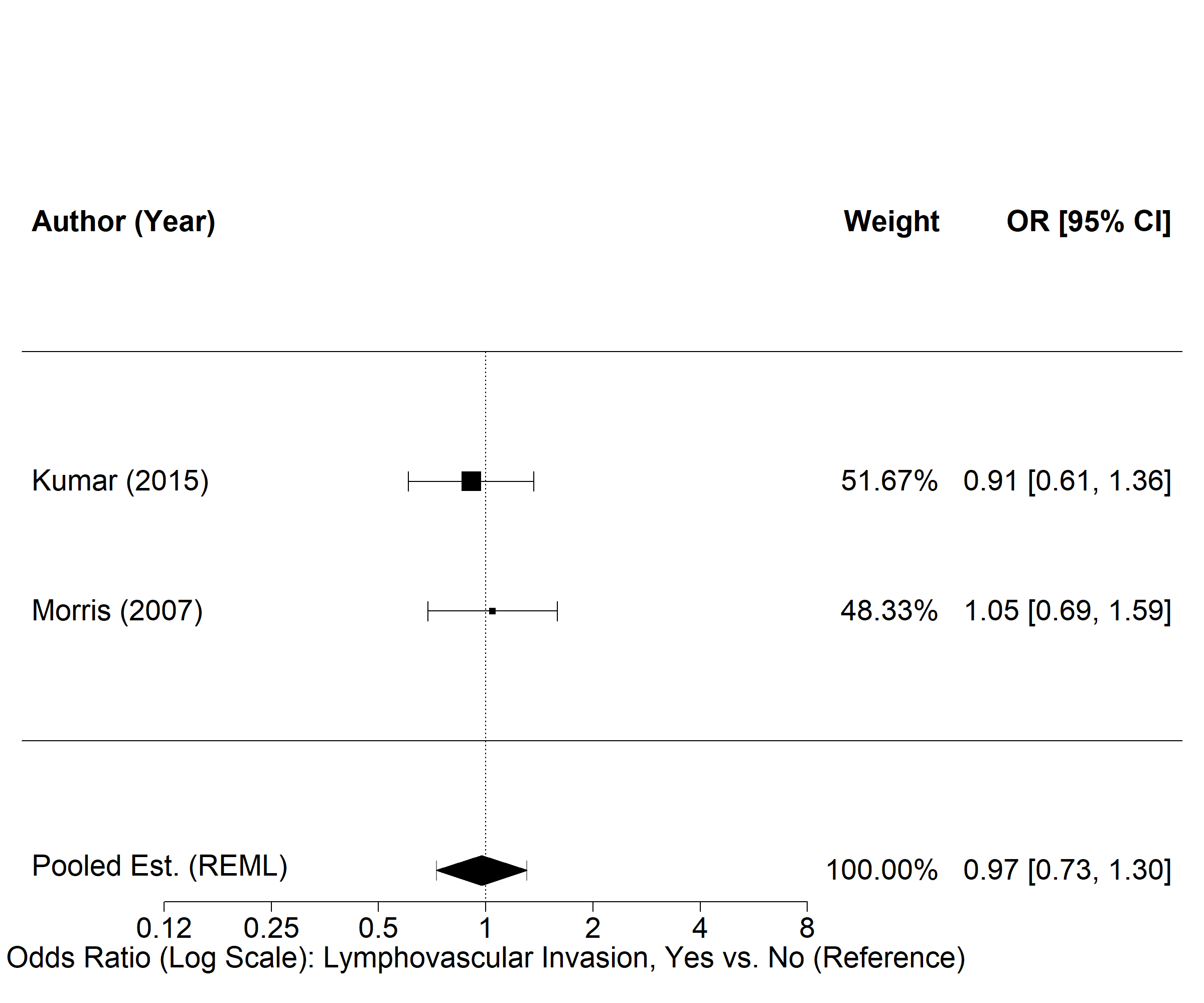


Figure S14. Meta-Analysis of the Association between Perineural Invasion and Chemotherapy Discontinuation among Stage II/III Colon Cancer Patients


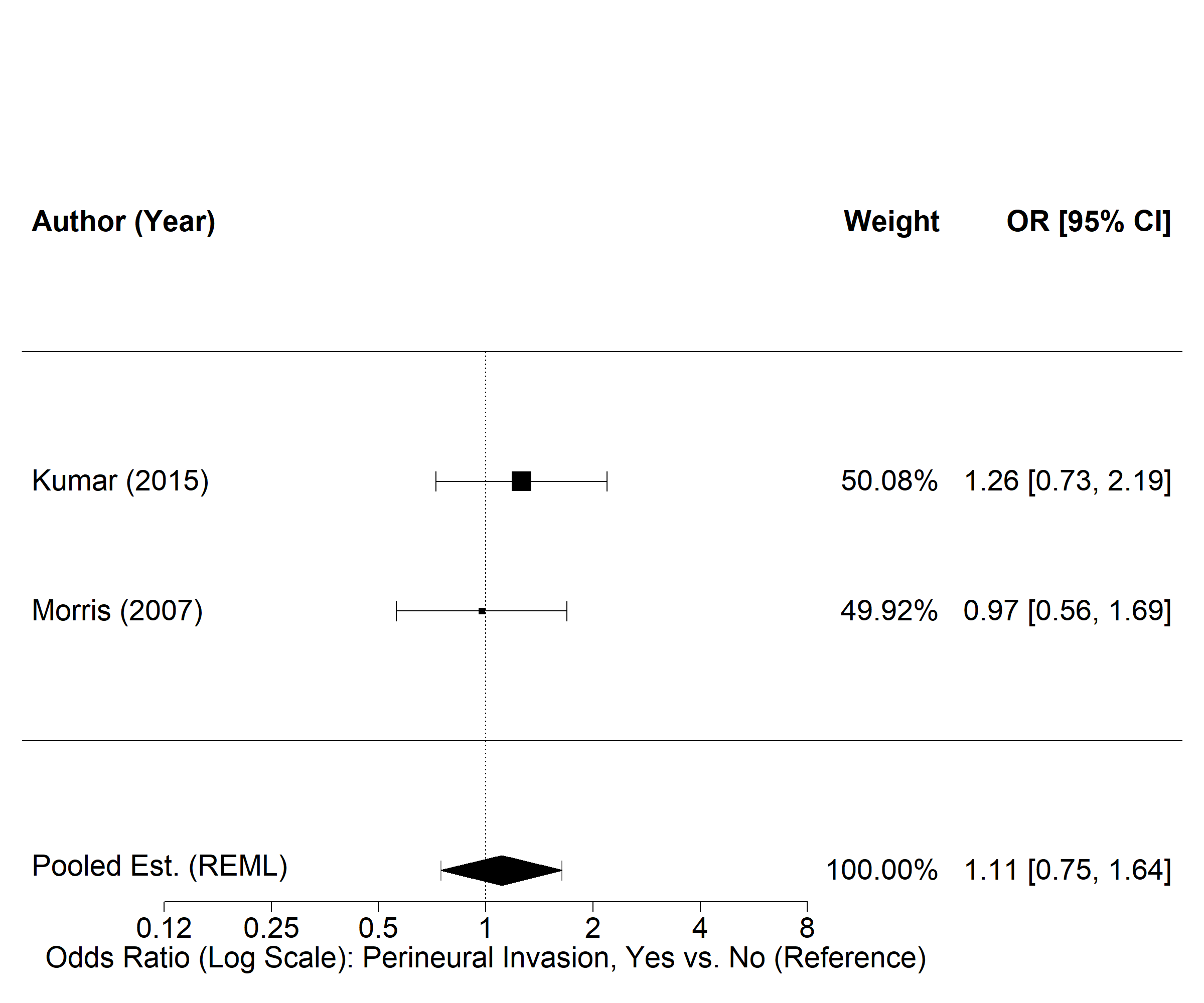


Figure S15. Meta-Analysis of the Association between Treatment Facility (Community vs. Academic) and Chemotherapy Discontinuation among Stage II/III Colon Cancer Patients


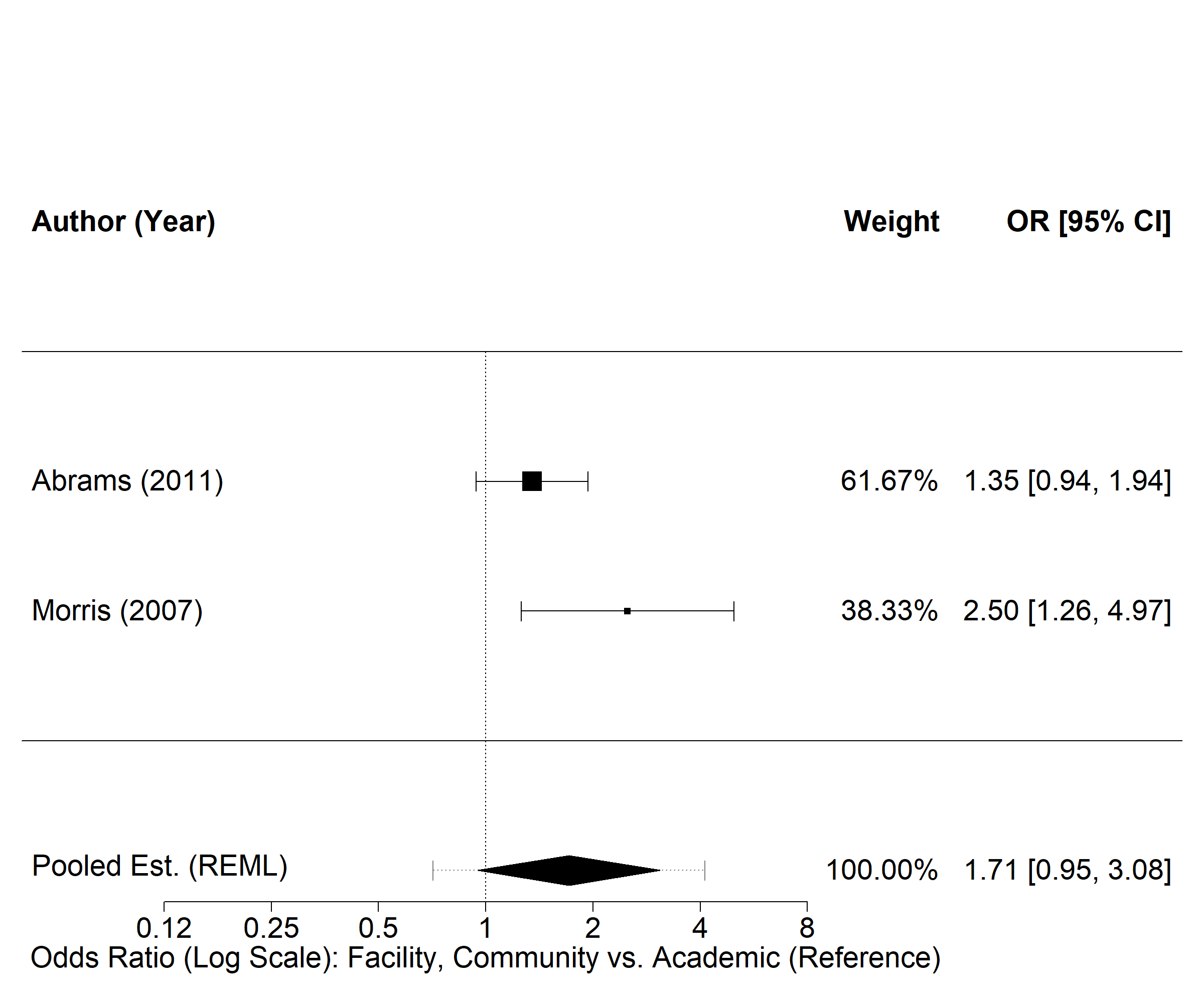


Figure S16. Meta-Analysis of the Association between Treatment Facility (Private vs. Academic) and Chemotherapy Discontinuation among Stage II/III Colon Cancer Patients


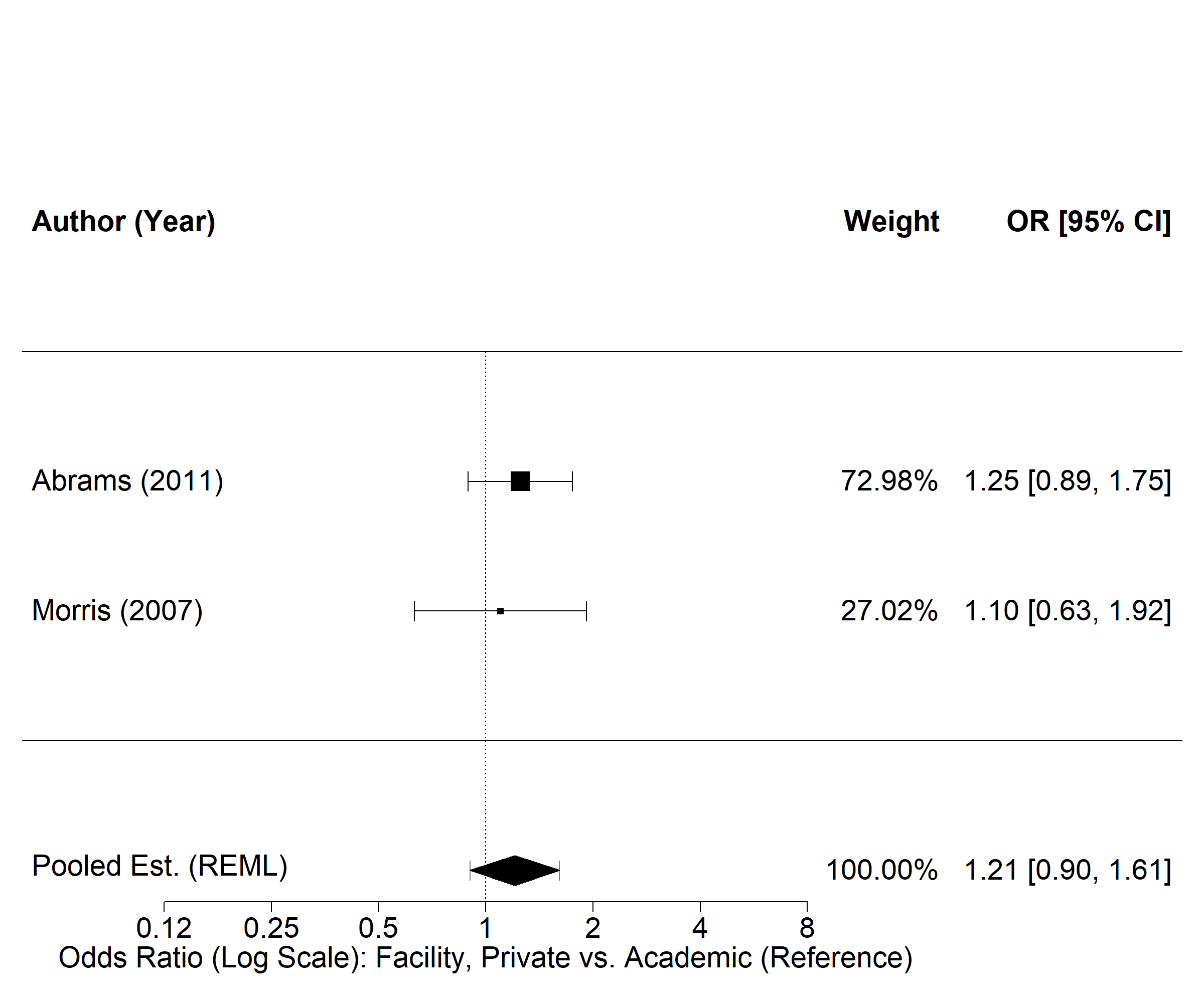


Figure S17. Meta-Analysis of the Association between Hospital Stay Duration and Chemotherapy Discontinuation among Stage II/III Colon Cancer Patients


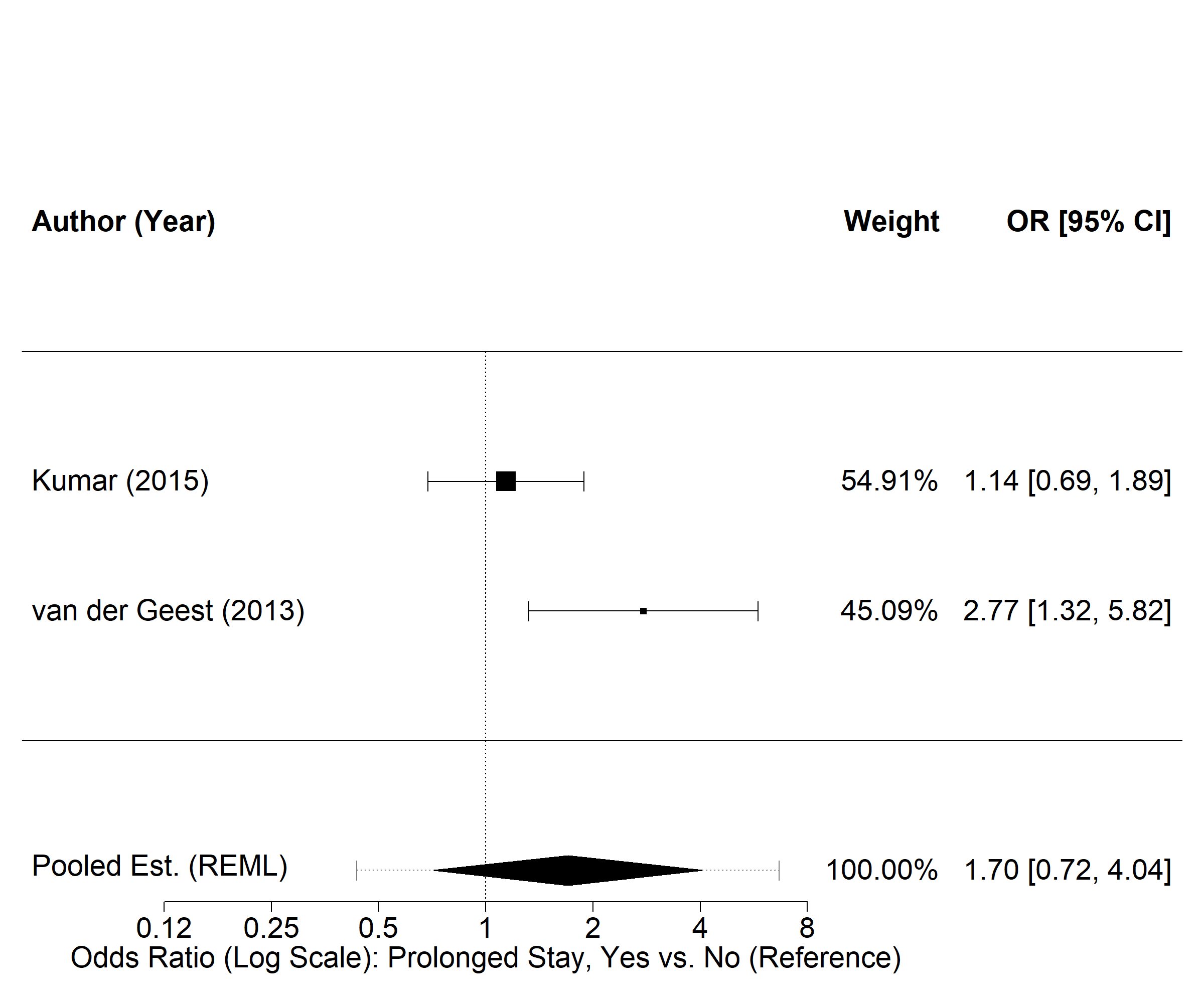


Figure S18. Funnel Plot to Assess Publication Bias among studies examining the association between Age and Chemotherapy Discontinuation among Stage II/III Colon Cancer Patients


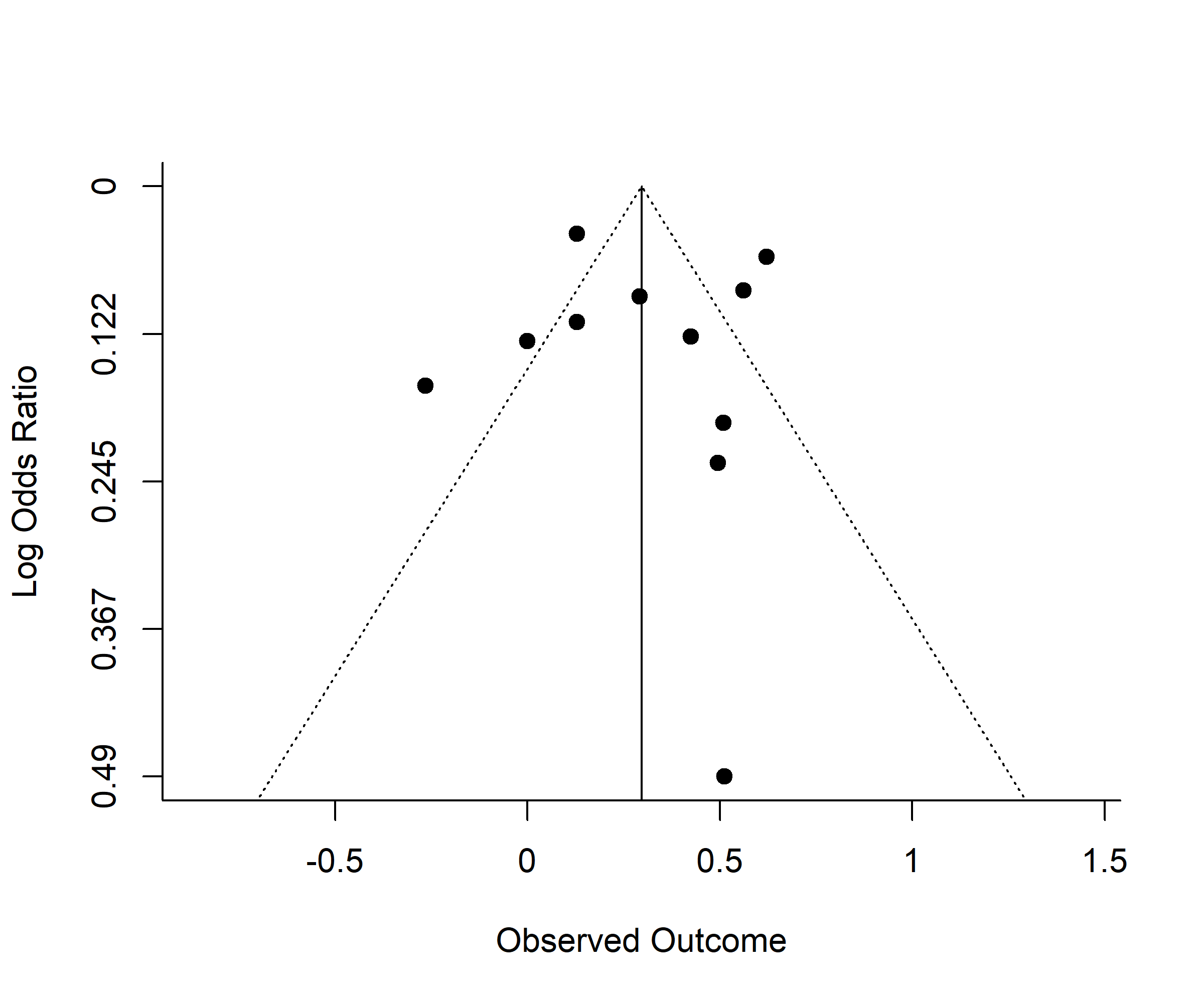

Supplement: Supplementary file 1 [file CAM4-9-1613-s001.docx]
